# Rapid generation of mouse model for emerging infectious disease with the case of severe COVID-19

**DOI:** 10.1371/journal.ppat.1009758

**Published:** 2021-08-11

**Authors:** Cheng-Pu Sun, Jia-Tsrong Jan, I-Hsuan Wang, Hsiu-Hua Ma, Hui-Ying Ko, Ping-Yi Wu, Tzu-Jiun Kuo, Hsin-Ni Liao, Yu-Hua Lan, Zong-Lin Sie, Yen-Hui Chen, Yi-An Ko, Chun-Che Liao, Liang-Yu Chen, I-Jung Lee, Szu-I Tsung, Yun-Ju Lai, Ming-Tsai Chiang, Jian-Jong Liang, Wen-Chun Liu, Jing-Rong Wang, Joyce Pei-Yi Yuan, Yin-Shiou Lin, Yi-Ching Tsai, Shie-Liang Hsieh, Chia-Wei Li, Han-Chung Wu, Tai-Ming Ko, Yi-Ling Lin, Mi-Hua Tao

**Affiliations:** 1 Institute of Biomedical Sciences, Academia Sinica, Taipei, Taiwan; 2 Genomics Research Center, Academia Sinica, Taipei, Taiwan; 3 Biomedical Translation Research Center, Academia Sinica, Taipei, Taiwan; 4 Graduate Institute of Microbiology, National Taiwan University, Taipei, Taiwan; 5 Solomont School of Nursing, Zuckerberg College of Health Sciences, University of Massachusetts Lowell, Massachusetts, United States of America; 6 Department of Biological Science and Technology, National Chiao Tung University, Hsin-Chu, Taiwan; 7 Institute of Cellular and Organismic Biology, Academia Sinica, Taipei, Taiwan; 8 Institute of Bioinformatics and Systems Biology, National Chiao Tung University, Hsinchu, Taiwan; Erasmus Medical Center, NETHERLANDS

## Abstract

Since the pandemic of COVID-19 has intensely struck human society, small animal model for this infectious disease is in urgent need for basic and pharmaceutical research. Although several COVID-19 animal models have been identified, many of them show either minimal or inadequate pathophysiology after SARS-CoV-2 challenge. Here, we describe a new and versatile strategy to rapidly establish a mouse model for emerging infectious diseases in one month by multi-route, multi-serotype transduction with recombinant adeno-associated virus (AAV) vectors expressing viral receptor. In this study, the proposed approach enables profound and enduring systemic expression of SARS-CoV-2-receptor hACE2 in wild-type mice and renders them vulnerable to SARS-CoV-2 infection. Upon virus challenge, generated AAV/hACE2 mice showed pathophysiology closely mimicking the patients with severe COVID-19. The efficacy of a novel therapeutic antibody cocktail RBD-chAbs for COVID-19 was tested and confirmed by using this AAV/hACE2 mouse model, further demonstrating its successful application in drug development.

## Author summary

Upon the emergence of new infectious disease, animal model becomes a pivotal tool for study of disease mechanism and development of therapeutics. In this study, we propose a versatile approach that allows rapid generation of mouse model for novel infectious disease once the receptor of the pathogen is identified. We demonstrated this approach by generating a mouse model for COVID-19 in a month’s time. These mice were capable of recapitulating severe COVID-19 in patients, and successfully applied in the development of a therapeutic antibody cocktail for the disease. This not only suggests the usefulness of this mouse model for the research on COVID-19, but also exhibit the utility of the proposed approach for establishing animal model for infectious disease.

## Introduction

In the past decades, new zoonotic virus infections continuously emerge and cause outbreaks in humans, such as coronavirus, alphavirus, filovirus, flavivirus and henipavirus [1]. Small animal models, especially the rodents, present a pivotal resource in the scenario for their roles in the study of disease mechanism and the development of vaccines and antiviral therapies. Nevertheless, sometimes rodents are not naturally permissive to certain human pathogens, and thus cannot be infected nor mimic the disease manifestation in human. In these cases, introducing the human receptor for such pathogen to the animals could be an efficient way to render them vulnerable to the infection and become a suitable disease model. For example, mouse models for SARS and MERS were generated in this manner [2,3]. To enhance the public health preparedness for emerging infectious diseases, it is critical to improve the method for animal model generation.

COVID-19 is the latest infectious disease that requires a special establishment of appropriate animal model. This disease, caused by novel severe acute respiratory syndrome coronavirus 2 (SARS-CoV-2), was first reported in December 2019 in Wuhan, Hubei province, China [4,5], and became a world-wide pandemic, resulting more than 174 million confirmed cases and 3.8 million deaths reported in more than 200 countries as of June 2021. SARS-CoV-2 is closely related to SARS-CoV with ~76% amino acid identity and a high degree of homology of the spike proteins (75%), which primarily determined the tropism of coronaviruses [6–8]. Several studies have demonstrated that SARS-CoV-2 utilizes the same entry receptor, human angiotensin-converting enzyme 2 (hACE2), as SARS-CoV [9–12]. However, despite the molecular similarities between the two viruses, SARS-CoV-2 displays a rather different pathology from SARS-CoV. For example, in the human patients, SARS-CoV-2 exhibits a dissemination advantage, but leads to milder lung lesions, less neutralizing antibody generation, and a lower fatality rate [13–15]. Therefore, to further our understanding of COVID-19 and to facilitate the development of antiviral therapeutics, a proper animal model was in urgent requirement.

Although several SARS-CoV-2 animal models have been described, such as monkeys, ferrets, and hamsters [13,16–20], mice are still the most appropriate animals for the convenience of small size, rapid breeding cycle, and the well-characterized immunological background. However, laboratory strains of mouse are shown to be non-permissive to SARS-CoV-2 infection due to the lack of viral entry receptor hACE2 [9,10,21]. At the time of writing, few groups have reported the mice permanently or transiently expressing hACE2. For instance, there are three transgenic mouse models using different promoters to drive permanent hACE2 expression, including a universal CMV enhancer/beta-actin (CB) promoter [22], epithelial cell-specific promoter (K18 or HFH4) [23,24], and the endogenous mouse ACE2 promoter [25]. Nevertheless, the production of hACE2 transgenic mice is time-consuming and the availability of these mice is yet limited. The transient hACE2 expression in mice can be achieved rather quickly by adenovirus (AdV)- or adeno-associated virus (AAV)-mediated transduction. The limit of AdV-based transduction is that AdV vector is prone to inducing immune responses upon the transduction, and hACE2 expression in transduced mice may only be maintained in the short term (less than one week) [26]. On the other hand, SARS-CoV-2 replication in mice sensitized by AAV/hACE2 transduction seems to be lower than that in other reported mouse models [27].

In this study, we describe an advanced strategy that allows the rapid establishment of a mouse model specific for SARS-CoV-2. By a combinatorial multi-route and multi-AAV-strain transduction of hACE2, its expression in our transduced mice was not just restricted to the lung, but also shown in various extrapulmonary organs, including heart and liver, which had been reported to be vulnerable to SARS-CoV-2 infection [28]. The transduced mice were susceptible to SARS-CoV-2, and showed significant weight loss, high levels of virus replication, as well as moderate to severe histopathology in lungs after the viral challenge. Other features seen in the patients with severe COVID-19 can also be observed in our mouse model, including neutrophilia, lymphopenia, and the similar pro-inflammatory cytokines and chemokines profiles. Upon SARS-CoV-2 infection, the AAV/hACE2-transduced mice we generated exhibit a high resemblance to the patients developing/recovering from severe COVID-19. This SARS-CoV-2 mouse model will not only advance our understanding of clinical manifestations of COVID-19 and patient’s condition during and post the recovery, but also benefit the development of the vaccine and antiviral interventions.

## Results

### Rapid establishment of mouse model for COVID-19 by multi-serotype AAV transduction

To date, almost all of the reported mouse models of SARS-CoV-2, including the those with hACE2 expression in lungs, remain inadequate to faithfully mimic COVID-19 in human [29,30]. Evidence suggests that COVID-19 is a systemic disease; in addition to the respiratory symptoms, there are also extrapulmonary manifestations, such as cardiac, gastrointestinal, renal, neurologic, and hepatic symptoms [31]. This may be linked to the wide distribution of hACE2 in human, including the lung, nasopharyngeal and oral mucosa, blood vessels, brain, gut, kidney, and liver [32]. Therefore, extrapulmonary organs in the animal model should also be rendered SARS-CoV-2-permissive to enable a close imitation of the disease, especially the severe cases. To quickly establish such a mouse model, an approach that combines AAV vectors with different tropisms and multiple administration routes was taken in this study.

**Fig 1:**
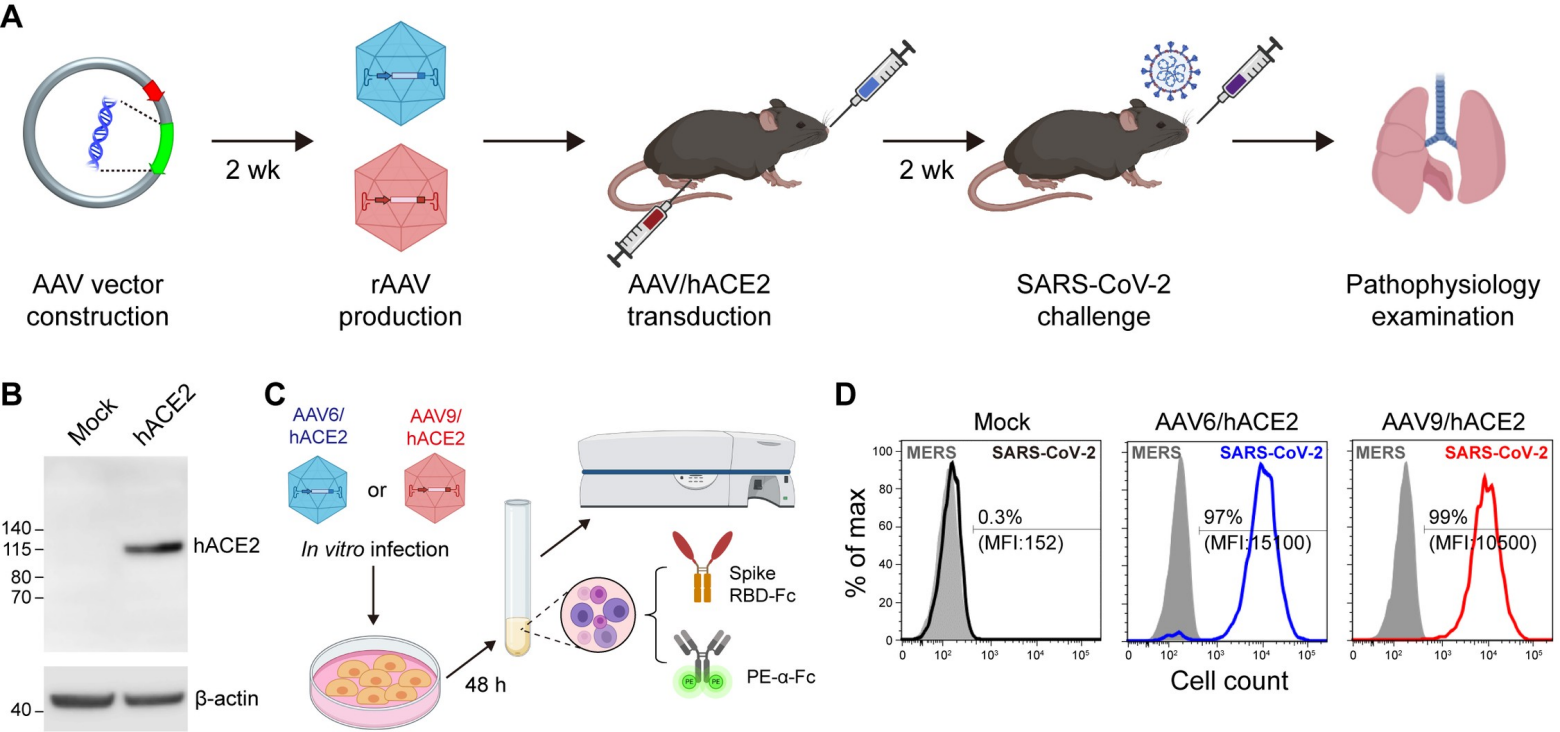
Establishment and examination of mouse model for COVID-19. (A) The workflow and time consumption of AAV vector construction and mouse model generation. (B) Western blot analysis of hACE2 expression in 3T3 cells mediated by the transfection of AAV expression vector carrying hACE2 gene. (C) The scheme depicts the procedure to examine the binding ability and specificity of hACE2 to SARS-CoV-2 RBD on AAV6/hACE2 or AAV9/hACE2 transduced 3T3 cells. (D) FACS analysis of hACE2 binding to SARS-CoV-2 RBD. MERS-CoV RBD serves as a negative control here. (MFI, mean fluorescent intensity).

The execution of this approach comprised the selection, construction, and administration of the AAV vectors (Fig 1A), as well as several examination steps for hACE2 expression and specificity (Fig 1B–1D); the whole process took about a month. AAV vectors are available in various serotypes that deliver the gene to different types of cell preferentially [33]. To transduce the aforementioned extrapulmonary organs with receptor expression and clinical manifestation in COVID-19 patients [31,34,35], we decided to use both AAV serotype 6 (AAV6) and serotype 9 (AAV9) for the delivery of hACE2. AAV6 is superior to other serotypes for the transduction of murine airway epithelial cell [36], and AAV9 shows a comprehensively high transduction efficacy in extrapulmonary organs [37,38]. hACE2 gene was cloned into a single-stranded AAV expression vector, downstream of a global CMV enhancer/beta-actin (CB) promoter. This hybrid promoter drives higher expression than other viral promoters alone, and leads to exceptional transgenic gene expression when used in combination with AAV vectors [39,40]. Before the packaging of pseudotyped AAV/hACE2 virions, the protein expression was confirmed by the western blot analysis of murine 3T3 cells transiently transfected with the constructed expression vector (Fig 1B). Subsequently, pseudotyped AAV6 and AAV9 carrying hACE2 were packaged and purified. To test the SARS-CoV-2-binding ability and specificity of AAV-delivered hACE2, AAV6/hACE2- and AAV9/hACE2-transduced 3T3 cells were incubated with mouse Fc-conjugated SARS-CoV-2 or MERS-CoV Spike receptor binding domain (RBD) recombinant proteins and then subjected to flow cytometry analysis (Fig 1C). Here, MERS-CoV RBD serves as a negative control, as the virus employs human dipeptidyl peptidase 4 (DPP4, also known as CD26) as its receptor and does not interact with hACE2 [41]. The result showed that AAV-delivered hACE2 could bind to the Spike RBD of SARS-CoV-2 specifically, but not to that of MERS-CoV (Fig 1D).

**Fig 2:**
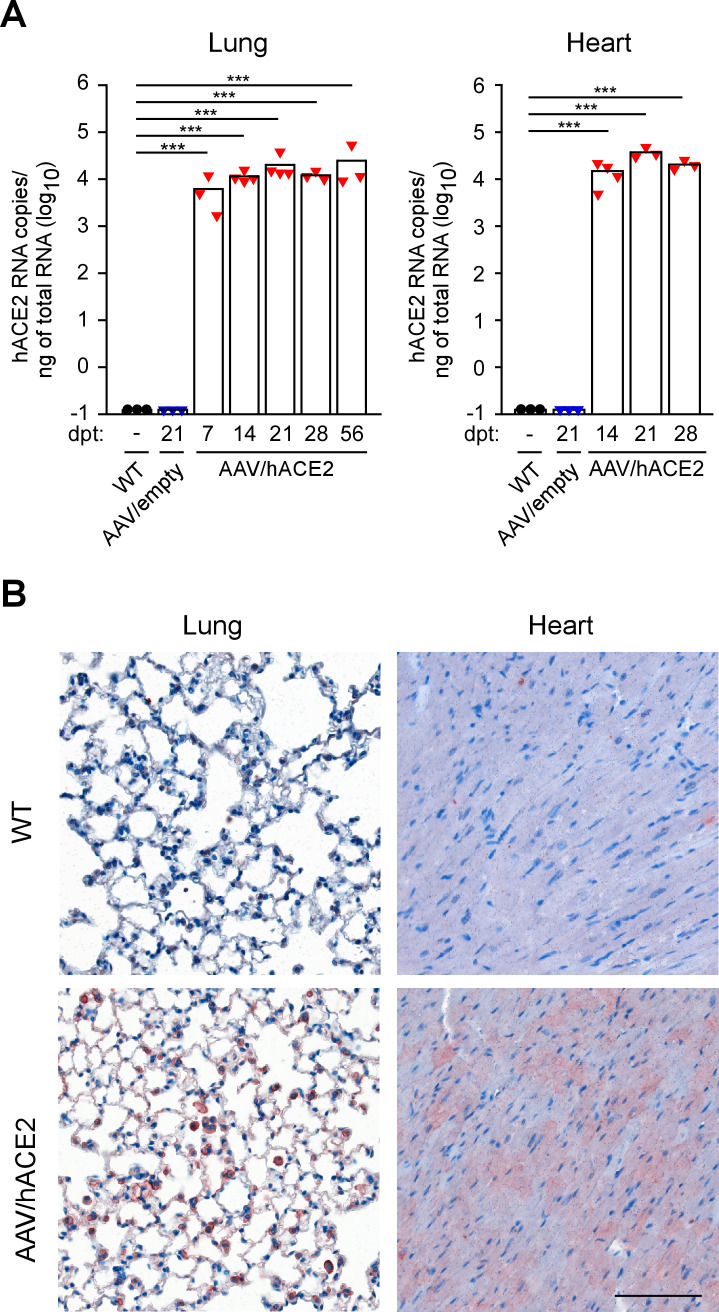
Expression of hACE2 in the lung and heart of mice transduced with AAV/hACE2. (A) RT-QPCR analysis of hACE2 expression level in the lung and heart of non-transduced wild type (WT, n = 3), AAV/empty-transduced (n = 3), and AAV/hACE2-transduced (n = 3 or 4) mice. P values were calculated by two-tailed unpaired Student’s t test (*, P < 0.05; **, P < 0.005; ***, P < 0.0005). (B) Representative immunohistochemical staining of hACE2 in the lung and heart of control (non-transduced) and AAV/hACE2-transduced mice. Tissues were analyzed at week 3 post AAV/hACE2 administration. Scale bar, 100 μm.

The selection of vector administration route is important for generating mouse model, as it has been reported to affect the efficacy of AAV-mediated gene transfer to specific organs [38,42]. Therefore, we compared the lung transduction efficiency of intratracheal (i.t.) and intranasal (i.n.) delivery in C57BL/6J (B6) mice by using AAV6/EGFP for transduction. Compared to i.n. administration, a drastic increase of EGFP-positive cells was observed in i.t. administered mice by fluorescence microscopy (S1 Fig). Additionally, the transduction efficiency of intraperitoneally (i.p.) administered AAV9 was also examined by using AAV9/EGFP. A prominent EGFP expression could be observed in the heart as well as the liver, and the kidney and the intestine were only partially positive of EGFP (S1 Fig). According to these results, we combined i.t. injection of AAV6/hACE2 and i.p. injection of AAV9/hACE2 to express hACE2 in mice, rendering them susceptible to SARS-CoV-2 infection. To establish hACE2 mice, B6 mice were administered with 3 x 10^11^ vg of AAV6/hACE2 and 1 x 10^12^ vg of AAV9/hACE2 through i.t. and i.p. route respectively. Consistent with the result of AAV/EGFP transduction test, strong hACE2 expression could be detected in the lung, heart, and also liver of AAV/hACE2-administered mice by RT-QPCR and immunohistochemical staining after one week post transduction (Figs 2A and 2B and S2A and S2B). Especially in the lung and heart, there were profound expressions of hACE2 (~10^4^ copies/ng of total RNA), comparable to those mouse models reported with high hACE2 level (e.g. K18-hACE2 mice, S2C Fig) [43]. Furthermore, this hACE2 expression was sustained for at least 28 weeks in the lung (S2D Fig), which was considered long-lasting for AAV-mediated gene expression in mice [38,44].

Before the mice were sent into animal biosafety level 3 laboratory (ABSL3) for further experiments, a pilot test was conducted to verify the susceptibility of AAV/ACE2 mice to SARS-CoV-2. The mice were intranasally infected with either vesicular stomatitis virus (VSV)-G pseudotyped virus or VSV-based pseudotyped SARS-CoV-2. Both pseudotyped viruses were replication-deficient and used red fluorescent protein (RFP) as a reporter. After 7 days of infection, RFP signals could be seen in the lung of AAV/hACE2 mice infected with pseudotyped SARS-CoV-2, but not in the non-transduced wild-type (WT) mice (S3 Fig). The observed RFP signals were at a similar level of that in the WT mice infected with VSV-G pseudotyped virus, which has a natural lung tropism [45]. This result gave a hint that this AAV/hACE2 mice were at least permissive to SARS-CoV-2 entry. Altogether, the data suggested that the AAV/hACE2 mice generated through proposed approach were ready for SARS-CoV-2 challenge.

### SARS-CoV-2 infection in AAV/hACE2 mice and resulted pathophysiological changes

**Fig 3:**
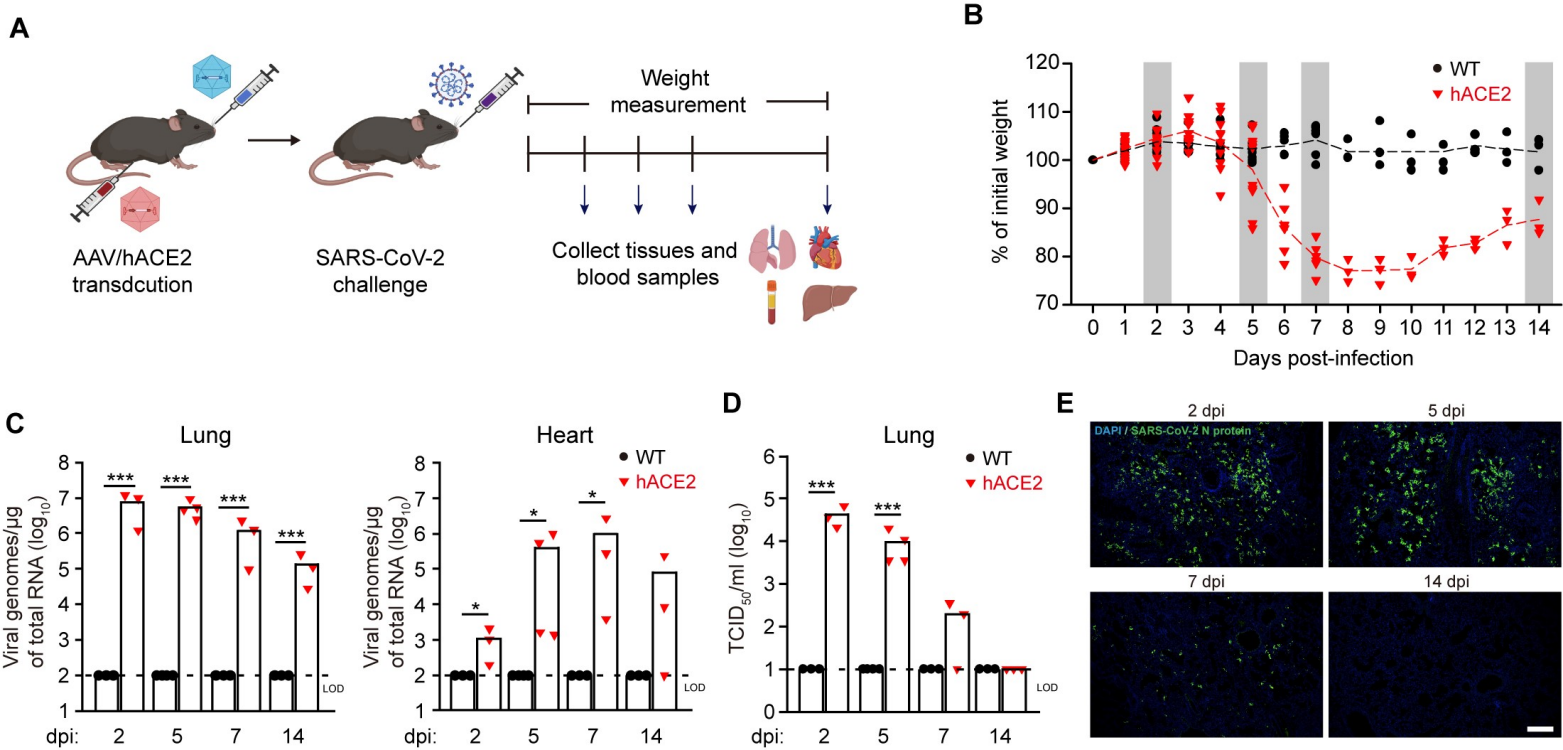
SARS-CoV-2 infection and body weight changes in AAV/hACE2 mice. (A) The experimental procedure of SARS-CoV-2 infection in AAV/hACE2 mice. (B) The weight changes of non-transduced wild-type (WT, black circle) and AAV/hACE2 (red triangle) mice challenged with SARS-CoV-2 at different days post-infection (dpi) (n = 13 at 0–2 dpi, n = 9 at 3–5 dpi, n = 6 at 6–7 dpi, and n = 3 at 8–14 dpi). Gray bars indicate the dates when lungs and blood samples are collected for further analyses. (C and D) The numbers of viral genomic RNA (C) and infectious virion (D) in the lung and heart of WT (black circle) and AAV/hACE2 (red triangle) mice at 2, 5, 7, and 14 dpi. Dashed lines indicate the limit of detection (LOD, 10^2^ viral genomes/μg of total RNA for subfigure c and 10^1^ TCID50/ml for subfigure d). Bars indicate mean values. P values were calculated by two-tailed unpaired Student’s t test (*, P < 0.05; **, P < 0.005; ***, P < 0.0005). (E) The lungs of infected AAV/hACE2 mice were collected at 2, 5, 7, 14 dpi, and the viral N proteins were detected by immunofluorescence staining (blue, DAPI; green, SARS-CoV-2 N protein). Scale bar, 200 μm.

First, the non-transduced WT and AAV/hACE2 mice were challenged with 1 x 10^5^ TCID_50_ of SARS-CoV-2 intranasally and monitored daily for the weight change (Fig 3A). A significant decrease in the body weight was found in infected AAV/hACE2 mice over a 14-day period, starting around day 5 post infection (p.i.) and achieving a peak of 23.9 ± 2.3% weight loss on day 7–9 p.i. (Fig 3B). Although the body weight of infected AAV/hACE2 mice gradually rebound after day 11 p.i., an average of 12 ± 2.6% loss of weight could still be observed on day 14 p.i. In parallel, the same experiment was also performed on the mice transduced with AAV6/hACE2 only via i.t. route and expressing hACE2 in the lung solely. In this case, only 5.7 ± 1.7% weight loss was observed on day 4–8 p.i., and then the body weight returned to the baseline (S4 Fig). This result is consistent with the previous report [27], and gave a hint on the importance of systemic expression of hACE2 in generating mouse model for COVID-19.

Next, the numbers of viral genomic RNA (gRNA) and infectious virion in the organs were measured on day 2, 5, 7, and 14 p.i. (Fig 3C and 3D). As compared with the control, the highest viral gRNA copy number was readily detected in the lung of infected AAV/hACE2 on day 2 p.i. (8.1 ± 5.9 x 10^6^ copies/μg of total RNA) and maintained at the same level till day 5 p.i. (7.2 ± 3.9 x 10^6^ copies/μg of total RNA) (Fig 3C). The high viral gRNA copy number persisted for at least two weeks, although a gradual reduction of it could be observed (1.3 ± 1.2 x 10^6^ copies/μg of total RNA on day 7 p.i. and 1.4 ± 0.1 x 10^5^ copies/μg of total RNA on day 14 p.i.). In addition, high viral gRNA copy number was also detected in the heart, suggesting that the heart was infected in our model. Nevertheless, the heart infection here exhibited a delayed kinetics and lower number of viral gRNA, compared to the infection in the lung. When it comes to the infectious virions generated during the infection, the viral titer measured in the lung of infected AAV/hACE2 mice also peaked on day 2 p.i. (4.2 ± 2.3 x 10^4^ TCID_50_/ml), similar to viral gRNA (Fig 3D). Interestingly, unlike the kinetics of viral gRNA, the titer of infectious virions decreased drastically after day 5 p.i. Compared to the peak titer, a 2-log reduction was observed on day 7 p.i. (1.9 ± 1.7 x 10^2^ TCID_50_/ml); and the virus titer was below the detection capability by the end of our experiment (day 14 p.i.). Jointly, the data indicates that the kinetics of SARS-CoV-2 infection in this AAV/hACE2 mice, in terms of viral gRNA and infectious virion levels, appears to be largely consistent with that in COVID-19 patients [46,47]. The same experiments were also performed on K18-hACE2 mice (SARS-CoV-2 challenge) and BALB/c mice (AAV/hACE2 transduction and SARS-CoV-2 challenge), and similar results were obtained (S5A and S5B and S5C and S5D Fig).

**Fig 4:**
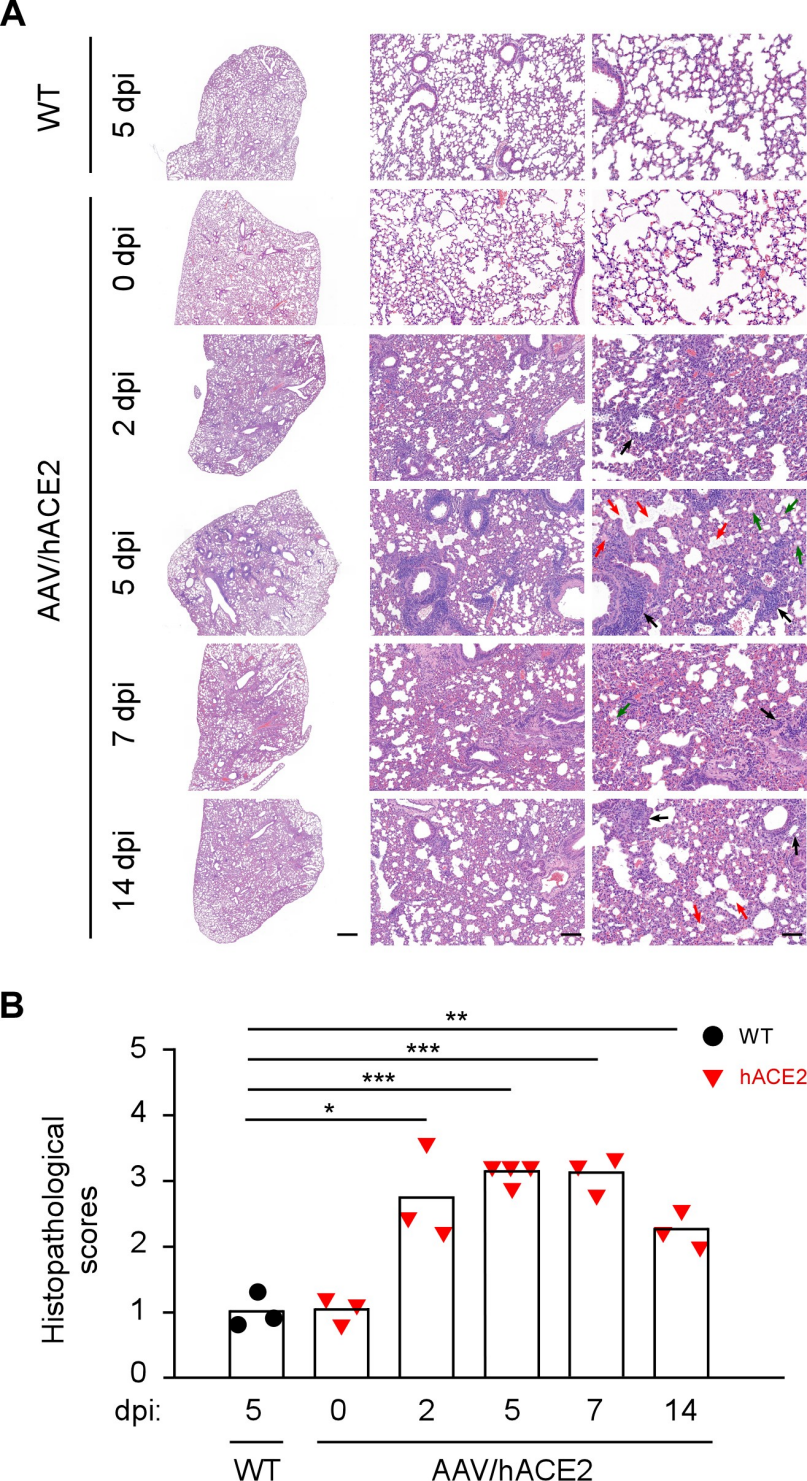
Pathophysiological changes in SARS-CoV-2-infected AAV/hACE2 mice. (A) The lungs of SARS-CoV-2-infected WT (5 dpi, n = 3) and AAV/hACE2 mice (0, 2, 5, 7, and 14 dpi, n = 3 or 4) were analyzed for pathophysiology by H&E stain examination. Peri-bronchial and peri-vascular inflammatory infiltration (black arrow), fibrin exudate (red arrow), and hyaline membrane formation (green arrow) can be observed in the lungs of challenged AAV/hACE2 mice. Micrographs are presented at low (left panel; scale bar, 1,000 μm), medium (middle panel; scale bar, 200 μm) and high (right panel; scale bars, 100 μm) magnifications. (B) Histopathological scores of the lung pathology in challenged WT (black circle) and AAV/hACE2 (red triangle) mice. Bars indicate mean values. P values were calculated by two-tailed unpaired Student’s t test (*, P < 0.05; **, P < 0.005; ***, P < 0.0005).

In addition to the viral replication, we also had a close look at the SARS-CoV-2-infected lung tissues of AAV/hACE2 mice. To minimize the variation among different lung lobes, the left lobe of lung was always used for the pathology examination. First, the infection status in the lung was inspected. As demonstrated by the immunofluorescence staining of viral nucleocapsid (N) protein, a large number of infected cells could readily be observed on day 2 p.i., which then greatly decreased on day 7 p.i. (Fig 3E). On day 14 p.i., although viral gRNA remained detectable (Fig 3C), pulmonary cells positive of N protein were rarely seen in the lung. This kinetic trend is similar to that of infectious virion production in the lung. Interestingly, the infection appeared to be evenly distributed through the whole lobe of lung on day 5 p.i. (S6A Fig), and immunofluorescence and immunohistological stainings further revealed that both alveolar cells and bronchial epithelial cells were infected on day 5 p.i. (S6B Fig). The infected lungs were then subjected to histopathological examination to see whether these mice acquired pneumonia. By the hematoxylin and eosin (H&E) staining, a variety of pathological changes were observed readily since day 2 p.i., such as peribronchial and perivascular to interstitial inflammatory infiltration with mononuclear cells, mainly lymphocytes and macrophages (Fig 4A). Furthermore, alveolar septa thickening, fibrin exudate, and hyaline membrane formation were also perceived. The pathophysiological condition of the lung was then rated by using a scoring system that evaluated the severeness of inflammation and diffuse alveolar damage [24,48]. According to the histopathological scores, an overall moderate to severe interstitial pneumonia was found in SARS-CoV-2-infected AAV/hACE2 mice (Fig 4B). The observed lung histopathology is largely similar to that in transgenic K18-hACE2 mice [49], and can also be observed in the infected AAV/hACE2 BALB/c mice (S5E Fig). Besides the lung, the heart was examined as it was shown to be infected by SARS-CoV-2 (Fig 3C) and clinically relevant. Although it was milder than what was reported for transgenic mice [23], heart pathology was observed in infected AAV/hACE2 mice. One mouse with the highest viral RNA titer (2.7 x 10^6^ copies/μg of total RNA) in heart on day 7 p.i. showed few occasions of vacuolar degeneration (S7 Fig). Lastly, since AAV pseudo-serotypes with hepatotropism (e.g. AAV9) is known to confer strong transgene expression in mouse liver upon i.p. administration (S1 Fig) [50] and there is also liver-related symptoms in COVID-19 patients [31], the liver of AAV/hACE2 mice was also subjected to analysis. It was infected but with comparably low amount of viral replication (maximum 4.6 ± 3.5 x 10^3^ vRNA copies/μg of total RNA after the challenge, S8A Fig), and there was no liver pathology observed (S8B Fig) in infected mice.

### Immunological changes in SARS-CoV-2 infection

**Fig 5:**
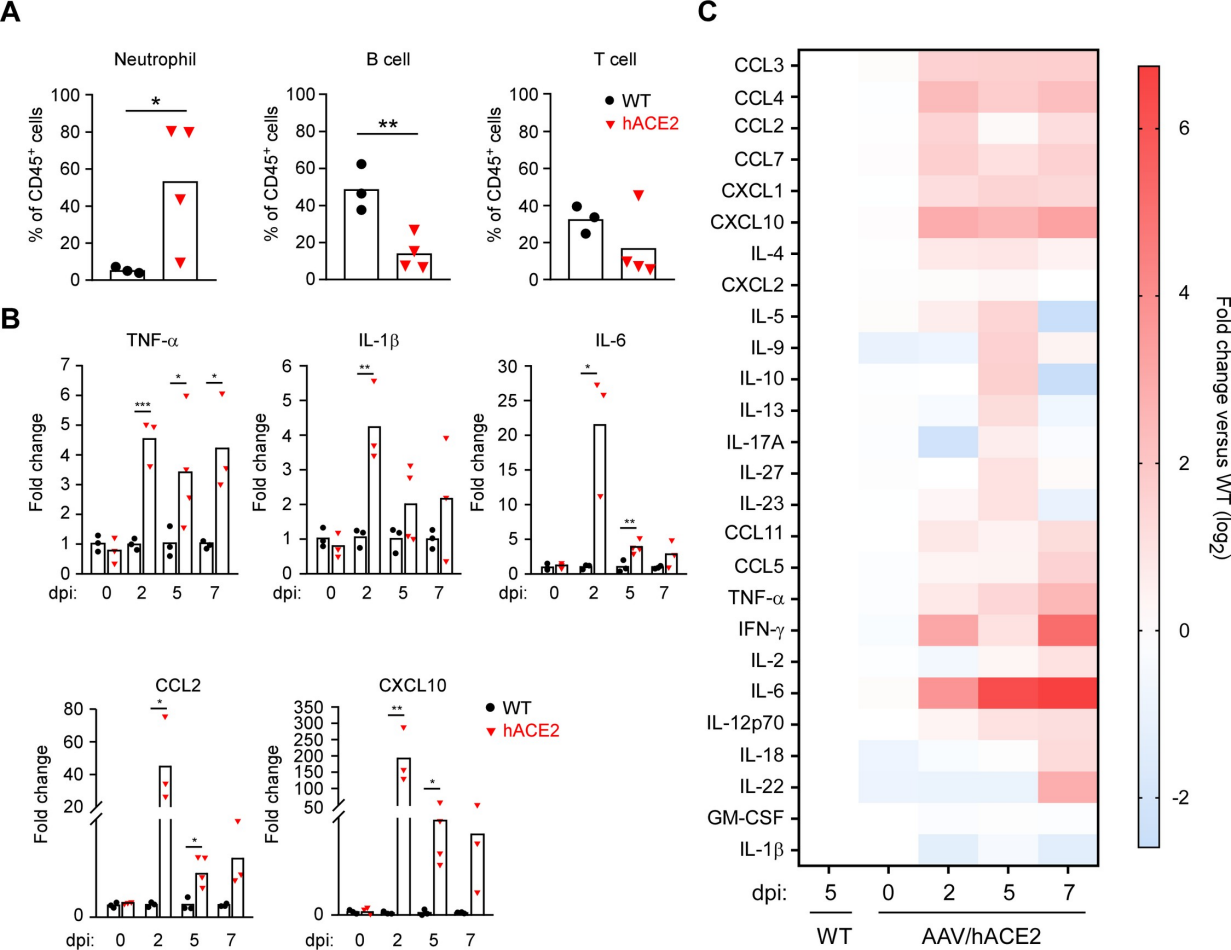
Immune profiles in SARS-CoV-2-infected AAV/hACE2 mice. (A) Composition analysis of peripheral mononuclear cells in the non-transduced wild-type (WT, black circle, n = 3) and AAV/hACE2 (red triangle, n = 4) mice on day 5 post-SARS-CoV-2 challenge. Percentage of neutrophil, B cell, and T cell subsets of total CD45^+^ cells were analyzed by FACS. (B) RNA expression level of proinflammatory mediators in the lung of WT (black circle) and AAV/hACE2 (red triangle) mice was analyzed by RT-QPCR at 0, 2, 5, and 7 days post-infection (dpi) (WT: n = 3; AAV/hACE2: n = 3 for 2 and 7 dpi, n = 4 for 5 dpi). The RNA level in challenged AAV/hACE2 mice is expressed as the fold-change compared with that in challenged WT control mice (mean values). (C) Cytokine/chemokine protein levels in the plasma of WT and AAV/hACE2 mice were analyzed by multiplex assay (WT: n = 3; AAV/hACE2: n = 3 for 0, 2, and 7 dpi, n = 4 for 5 dpi). For each gene, the fold change was defined as compared with the control (SARS-CoV-2-challenged WT mice). The data is presented in the format of heat map (original data can be seen in S9 Fig).

It has been reported that excessive inflammatory responses in SARS-CoV-2 infection was associated with disease severity and death in COVID-19 patients [51–53]. Therefore, multiple inflammatory markers in SARS-CoV-2-infected AAV/hACE2 mice were measured to examine the resemblance between the mouse model and COVID-19 patients. Neutrophil-to-lymphocyte ratio (NLR) serves as a marker of subclinical inflammation and is an important feature and predictor of severe COVID-19 [54–57]. We thus analyzed the composition of CD45^+^ immune cells in the peripheral blood of AAV/hACE2 mice by flow cytometry. On day 5 p.i., strong neutrophilia (9.9-fold increase) and lymphopenia (3.4-fold decrease for B cell and 1.9-fold decrease for T cell) were observed (Fig 5A). This resulted in a 19-fold increase of NLR (WT mice: 0.16, AAV/hACE2 mice: 3.1), which correlated with the occurrence of severe COVID-19 in infected mice (Fig 4) [58]. Subsequently, to further determine whether SARS-CoV-2 infection in AAV/hACE2 mice model could also induce proinflammatory mediators as shown in severe COVID-19 patients [4,59,60], RNA levels of TNF-α, IL-1β, IL-6, CCL2, and CXCL10 in mice lung homogenates were measured. As compared to the non-transduced WT mice, AAV/hACE2 mice showed a rapid and robust induction of these proinflammatory mediators in the lung after SARS-CoV-2 infection, with their RNA expressions all peaked on day 2 p.i (Fig 5B). Nevertheless, the RNA levels of IL-6, CCL2 and CXCL10 decreased after 5 days of infection, especially IL-6 that went down with more than 5-fold reduction compared to that on day 2 post infection. Additionally, a multiplex assay was performed to measure the protein level of circulating chemokines and cytokines in the plasma (Figs 5C and S9). Several chemokines were induced quickly in the early phase (day 2 p.i.) and maintained at a rather high level through the first week of infection, including CCL2, CCL3, CCL4, CCL7, CXCL10, IL-6, IFN-γ, CCL11, CCL5, TNF-α, CXCL1, IL-4, IL-12p70. Some other immune mediators also increased but with a delayed kinetics: IL-5, IL-9, IL-10, IL-13, IL-17, IL-23, and IL-27 induction peaked on day 5 p.i., while IL-2, IL-18, and IL-22 peaked on day 7 p.i. Interestingly, contradictory to the results obtained from the lung samples, the protein level of IL-6 was maintained at the high-level all the time in the circulation. In human patients, significantly higher plasma level of IL-6 was seen in mortal cases [61]. Jointly, the data suggest that SARS-CoV-2-induced immune responses in AAV/hACE2 mice, in terms of inflammation and chemokine/cytokine regulation, closely mimics the immunological profiles of severe COVID-19 patients.

### Development of therapeutic antibodies for treating COVID-19 by using AAV/hACE2 mice

**Fig 6:**
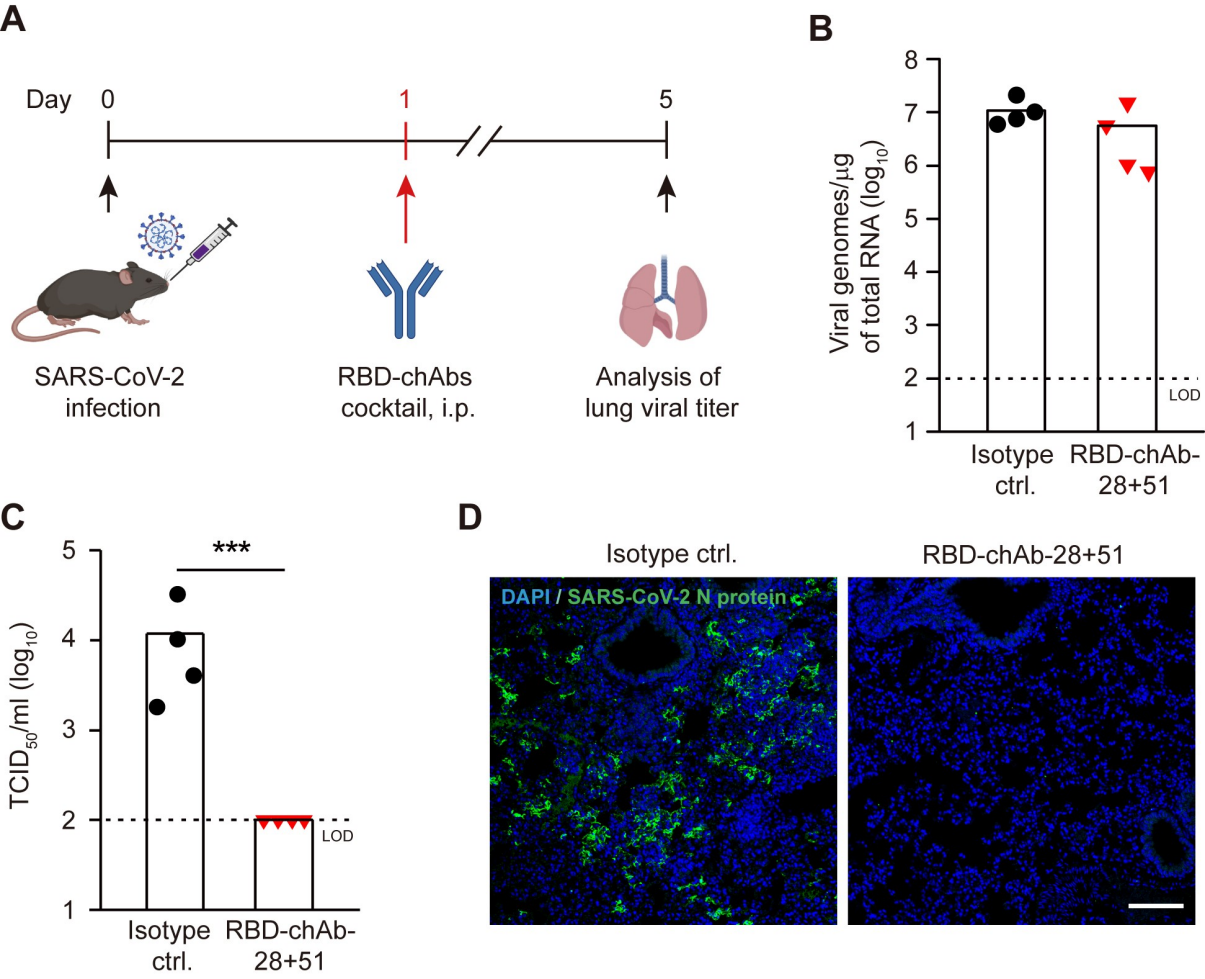
Evaluation of *in vivo* efficacy of the therapeutic RBD-chAbs cocktail. (A) The experimental procedure of evaluating RBD-chAbs cocktail efficacy in AAV/hACE2 mice. RBD-chAbs cocktail was given to the mice on day 1 post-infection. (B) The numbers of viral genomic RNA in the lungs of AAV/hACE2 mice treated with isotype control (black circle, n = 4) or RBD-chAbs cocktail (red triangle, n = 4) were measured by RT-QPCR. The dashed line indicates the limit of detection. (C) The titer of infectious virions in the lungs of AAV/hACE2 mice treated with isotype control (black circle) or RBD-chAbs cocktail (red triangle) were measured by TCID_50_ analysis. Dashed lines indicate the limit of detection (LOD, 10^2^ viral genomes/μg of total RNA for subfigure B and 10^2^ TCID_50_/ml for subfigure c). Bars indicate mean values. P values were calculated by two-tailed unpaired Student’s t test (*, P < 0.05; **, P < 0.005; ***, P < 0.0005). (D) Visualization of SARS-CoV-2 infection in the lungs of AAV/hACE2 mice treated with isotype control or RBD-chAbs cocktail (blue, DAPI; green, SARS-CoV-2 N protein). Scale bar, 100 μm.

To demonstrate the application of established mouse model in the research on therapeutics for COVID-19, AAV/hACE2 mice were employed to examine the efficacy of an antibody cocktail that comprises two chimeric anti-SARS-CoV-2 spike RBD monoclonal antibodies (RBD-chAb-28 and RBD-chAb-51). These RBD-specific chimeric antibodies could effectively block SARS-CoV-2 infection in Vero-E6 cells, with plaque reduction neutralization test (PRNT50) values lower than 30 ng/ml (Su *et al*., manuscript accepted). To further verify their *in vivo* efficacy, the AAV/hACE2 mice were infected with SARS-CoV-2 (1 x 10^5^ TCID_50_) and given an i.p. injection of RBD-chAbs cocktail (20 mg/kg of mouse) or isotype control on day 1 p.i. (Fig 6A). On day 5 p.i., the mice were sacrificed, and the numbers of viral gRNA and infectious virions in the lungs were measured. The treatment led to approximately 2-fold decrease of the viral gRNA copy number in the infected mice lungs (P = 0.083) (Fig 6B), and further reduced the number of generated infectious virions to below the limit of detection (LOD) (Fig 6C). In addition, we also analyzed the expression of viral N protein and subgenomic (sg) viral Envelope (E) gene, as well as the active virus replication (indicated by negative-strand vRNA) [62]. In the control, numerous pulmonary cells were infected and positive of SARS-CoV-2 N protein as shown by the immunofluorescence staining (Fig 6D), and the lung homogenate contained 2.1 ± 2.0 x 10^7^ sg E RNA copies/μg of total RNA (S10A Fig) and 1.9 ± 1.5 x 10^5^ copies of negative-strand vRNA/μg of total RNA (S10B Fig). Yet in the infected mice treated with RBD-chAbs cocktail, there is a drastic decrease of infected cells in the lung, with only few cells positive of N proteins sporadically observed (Fig 6D). Furthermore, there were significant reduction of E sg vRNA expression (about 6.5-fold decrease to 3.2 ± 3.9 x 10^6^ copies/μg of total RNA, S10A Fig) and active virus replication (about 5.3-fold decrease to 3.5 ± 5.0 x 10^4^ copies of negative-strand vRNA/μg of total RNA, S10B Fig). Altogether, the results indicate that RBD-chAbs cocktail could efficiently restrain the spreading of SARS-CoV-2 in AAV/hACE2 mice, possibly via suppressing viral replication and/or virion formation.

## Discussion

The establishment of proper animal models is pivotal in the research on infectious diseases, especially the study of complex immune response to the infections and the disease progression *in vivo*. It serves as a bridge between *in vitro* discoveries and clinical findings and promotes the development of preventional and therapeutic medications. Here, we report an advanced strategy that rapidly renders mice vulnerable to the latest SARS-CoV-2 infection and capable of mimicking severe COVID-19 by combining pulmonary and systemic delivery of AAV/hACE2 vectors with different tissue tropisms. Currently, several hACE2-expressing mouse models for COVID-19 have been reported, generated via transgenic approach or viral vector-mediated gene transduction. Among these models, transgenic mice with global hACE2 expression driven by cytokeratin-18 (K18) promoter (K18-hACE2 mice) demonstrates the most relentless disease development. Upon SARS-CoV-2 infection, K18-hACE2 mice loses approximately 25% of weight and shows marked viral replication and pathology in the lung, systemic infection and pathology in multiple organs, pulmonary immune response resembling that in severe human patients, and even mortality [63]. Nevertheless, the seeming correlation between the mortality of infected K18-hACE2 mice and brain infection raises the apprehension of using this mouse model for pathogenesis study, as brain infection is not the major cause of death in human patients with COVID-19 [64]. Furthermore, the generation of transgenic mice is time-consuming, typically requiring more than half a year of work. The viral vector-mediated gene delivery provides an alternative way of quickly introducing the viral receptor precisely to clinically relevant organs. For COVID-19 mouse model, pulmonary AdV- and AAV-transduction were employed to express hACE2 specifically in mice lungs [26,27,65]. The resulted mouse models are susceptible to SARS-CoV-2 and display similar pulmonary pathology and immune responses upon the infection, although the viral replication in the lung (S5 Fig) and body weight loss are slightly gentler compared to those in infected K18-hACE2 mice [66]. Furthermore, viral vector-mediated transduction is flexible and can be applied to transduce not only wild-type but also genetically modified mice, such as immunodeficient mice, and thus benefit the study of immune responses in COVID-19 [27]. However, the infection and pathology that are actually systemic in COVID-19 patients may be overlooked in reported transduced-mouse models due to the restrained pulmonary hACE2 expression, which renders these models less ideal. Therefore, we set out to advance the transduction approach to enable the rapid generation of mouse model for COVID-19 that possesses desired systemic organ susceptibility and closely mimics the severe disease.

In this study, AAV vector was used to transduce mice for its low immunogenicity (for both innate and adaptive immunities) and ability to raise sustainable protein expression (for at least 120 days) [44], which would benefit the subsequent studies of the viral infection and corresponding host responses. The combinatory AAV/hACE2 administration strategy (i.t. injection of AAV6 and i.p. injection of AAV9) employed here indeed resulted in exceptionally high and enduring hACE2 expression in mice lungs (lasting for at least 7 months, S2D Fig). It confers SARS-CoV-2 susceptibility specifically to target organs, which are reported to be infected and show pathology in COVID-19 patients (Fig 2) [4]. With the systemic SARS-CoV-2 infection, the generated AAV/hACE2 mice demonstrates significant body weight loss after day 5 post-infection, high viral burden in the lung, moderate to severe interstitial pulmonary pneumonia, as well as the concomitant increased NLR and high levels of pro-inflammatory cytokines/chemokines in lungs and peripheral blood (Figs 3–5 and S5–S9). These severity and disease kinetics displayed in our AAV/hACE2 mice are comparable to the aforementioned K18-hACE2 mice and closely mimics severe COVID-19 in human patients, yet the producing time for our moues model is much shorter than that for K18-hACE2 mice (less than one month and several months to one year, respectively). Although the fatality upon SARS-CoV-2 infection was not observed in our AAV/hACE2 mice, they remain an excellent model of non-fatal severe COVID-19, especially competent for the mechanistic studies of immune response, pathology, post-infection syndrome, and the recovery. For instance, the immune response to SARS-CoV-2 infection and its kinetics in infected mice were carefully examined to validate the potential of our AAV/hACE2 mice in related study of COVID-19. Despite the lymphopenia and general trend of promoted inflammation that are important features of severe COVID-19 [56,67], the subtle changes in individual cytokines/chemokines in infected AAV/hACE2 mice are also comparable to those in patients, such as the increase of TNF-α, IL-6, CCL2, and CCL3 in the circulation (Fig 5) [4,60,68]. The results imply that our mouse model can be useful for studying critical immune-related issues about COVID-19, such as identifying the primary source of cytokine storm in patients, or whether the tissue damage and organ failure of patients are caused by SARS-CoV-2 itself or the infection-induced cytokine storm. More interestingly, the immunologic kinetic in our SARS-CoV-2-infected mice largely resembles that in COVID-19 patients recovering from severe infection. In these AAV/hACE2 mice, pro-inflammatory cytokines/chemokines, such as IL-6 and CXCL10, were triggered from day 2–5 p.i. Subsequently on day 7 p.i., IFN-γ elevated and the expression of TNF-α and IL-2 received a further boost, possibly led by the monocytes, macrophages and T cells attracted to the infection sites due to the continuous cytokine/chemokine stimulation. As from day 7–14 p.i., these cytokines may induce SARS-CoV-2-specific adaptive immune responses, which will eventually lead to virus elimination. This scenario is also observed in recovering COVID-19 patients [69], and the similarity in AAV/hACE2 and human patients suggests that our model may serve as a good tool for further investigation on tissue damage and repair after viral clearance, which is important in the post-COVID-19 era.

Another valuable application of animal model for infectious disease is to assess the efficacy of vaccines and therapeutics *in vivo*. Here, we demonstrate the competence of a novel therapeutic RBD-chAbs cocktail to treat COVID-19. The cocktail consists of two strongly neutralizing chAbs that target distinct sites of SARS-CoV-2 RBD and are selected by hybridoma screening (Su *et al*., manuscript accepted). In the SARS-CoV-2-infected AAV/hACE2 mice treated with RBD-chAbs cocktail, although the level of viral genomic RNA remained high in the lung on day 5 p.i., there was no infectious virions detected. This phenomenon resembles the outcome of oseltamivir (brand name: *Tamiflu*) treatment in animals infected with influenza virus [70]. Oseltamivir is an inhibitor targeting the neuraminidase of influenza virus and can block the viral spread by inhibiting the release of progeny virions from infected cells. The data give a hint that RBD-chAbs cocktail may intervene SARS-CoV-2 infection in a similar fashion as oseltamivir does to influenza virus. We believe that RBD-chAbs cocktail could serve to treat COVID-19 patients or as a prevention medicine for personnel in high-risk environment.

Altogether, the results show that our AAV/hACE2-transduced mice are susceptible to SARS-CoV-2, and closely mimic the conditions of patients experiencing/recovering from severe COVID-19. This SARS-CoV-2 mouse model is applicable in the development of antiviral therapeutics. With that being said, there is room for improvement of our mouse model in the future. Firstly, neurological symptom is an important feature of COVID-19, literally the loss of taste or smell, and cannot be addressed by the current AAV/hACE2 mice due to the lack of hACE2 expression (and resulted absent viral replication) in brain (S11A Fig). The latest evidence also suggests that there is brain invasion of SARS-CoV-2, albeit it is sporadic and not the major cause of death in COVID-19 patients [71]. To enable the study of neurological deficit in COVID-19, currently available AAV vectors with various neuronal tropism (e.g. AAV9, AAV-PHP.S, AAV-PHP.eB, AAV-PHP.B [72], AAV rhesus(rh.)10, AAVrh39, and AAVrh43 [73,74]) and administration routes (such as intrastriatal, intrathalamic, intracerebroventricular, and intrathecal) [75], can be used to selectively transduce specific regions in the mouse brain that are clinically relevant. This may allow SARS-CoV-2 neuron invasion in the mice but avoid excessive brain infection and corresponding mortality. Secondly, in our current transducing procedure, i.t. injection was selected to transduce respiratory system to maximize the hACE2 expression in the lung and simplified the AAV administration procedure. In this case, upper respiratory tract, especially the nasal turbinate, may be circumvented or under-transduced for hACE2 expression. Nevertheless, upper respiratory tract is the primary target of incoming SARS-CoV-2 and critical for the progeny virion transmission [76]. Therefore, intranasal administration of AAV6/hACE2 could be included in the future making of AAV/hACE2 to enable the viral replication in the upper respiratory tract, such as the nasal turbinate (S11B Fig). Such mouse model will be beneficial for the study of SARS-CoV-2 transmission. Lastly, since AAV vector is flexible in multi-gene delivery, the co-expression of non-canonical SARS-CoV-2 receptors and protein related to viral entry, such as CD147 and serine protease TMPRSS2 [9,77], can be attempted to enhance mice susceptibility to infection. With these modifications, the mouse model will better recapitulate COVID-19 upon SARS-CoV-2 infection and provide a greater platform for the future studies.

Finally, the combinatory AAV-transduction approach demonstrated in this study has the full potential to be generalized and applied to establish mouse model for any infectious diseases. In the recent decades, new viruses/viral strains and zoonotic diseases have brought, and will continue bringing, great threaten and burden to the human society. When an emerging infectious disease appears, the corresponding animal model will be in urgent demand for research on pathology and therapeutic development. Regardless of the viral tropism, the chance is high that one can chose from the wide variety of AAV vectors and various administration routes to deliver the gene of identified viral receptor to the clinically relevant organs in mice. Furthermore, the time required for generating the mouse model by proposed versatile approach is extremely short, compared to the making of transgenic mice. Following the workflow depicted in Fig 1, the animal model for the emerging infectious disease can be expected in one month after the pathogen’s receptor is identified, with 1–2 weeks for preparing the AAV vectors and 2 weeks for receptor expression. The rapid and almost immediate establishment of mouse model can make substantial contribution to the understanding of clinical manifestations of emerging infectious disease, as well as the development of vaccine and antiviral treatments. Even if it is just one day earlier to obtain such knowledge and cure for the infectious diseases, it will enhance our chance in battling the yet to come viruses.

## Materials and methods

### Ethics statement

All mouse works were conducted in accordance with the “Guideline for the Care and Use of Laboratory Animals” as defined by the Council of Agriculture, Taiwan. Mouse work was approved by the Institutional Animal Care and Use Committee of Academia Sinica (protocol ID: 20-05-1471 and 19-07-1330). The Institutional Biosafety Committee of Academia Sinica approved work with infectious SARS-CoV-2 virus strains under BSL3 conditions. All sample processes were conducted according to “Interim Laboratory Biosafety Guidelines for Handling and Processing Specimens Associated with Coronavirus Disease 2019 (COVID-19)” recommended by CDC.

### Animals

C57BL/6J and BALB/c mice were purchased from the National Laboratory Animal Center (Taipei, Taiwan). B6.Cg-Tg(K18-ACE2)2Prlmn/J transgenic mice (stock number: 034860) were purchased from the Jackson Laboratory (ME, USA). All mice were maintained as small breeding colonies in a specific pathogen-free environment in the animal facilities of the Institute of Biomedical Sciences, Academia Sinica. All experimental procedures were reviewed and approved by the Animal Care and Use Committee of Academia Sinica.

### Cells and viruses

The monkey kidney epithelial Vero-E6 cells, murine embryonic fibroblast 3T3 cells, and human embryonic kidney 293T cells were grown at 37°C under 5% CO_2_ in the growth medium [Dulbecco’s modified Eagle’s medium (DMEM, Gibco, Thermo Fisher Scientific, MA, USA) supplemented with 10% fetal bovine serum (FBS) and 1% penicillin/streptomycin (P/S, Gibco, Thermo Fisher Scientific)]. The transfection of 3T3 cells was performed by using calcium phosphate.

The SARS-CoV-2 used in this study, the clinical isolate TCDC#4 (hCoV-19/Taiwan/4/2020), was obtained from Taiwan Centers of Disease Control (CDC) and propagated on Vero-E6 cells in DMEM supplemented with 2% FBS and 1% P/S. The propagation of SARS-CoV-2 was conducted in the biosafety level 3 (BSL3) facility in the Institute of Biomedical Sciences, Academia Sinica. Vesicular stomatitis virus (VSV) G pseudotyped virus and VSV-based pseudotyped SARS-CoV-2 were produced by transient transfection of 293T cells using X-tremeGENE HP DNA Transfection Reagent (Roche AG, Basel, Switzerland) with vector plasmid (pLAS2w.RFP-C.Ppuro from National RNAi Core Facility Platform, Academia Sinica, or pGL4.18 CMV-Luc from Addgene, MA, USA), lentiviral helper plasmid (pCMV-dR8.2 dvpr, Addgene), and envelope plasmid (pCMV-SARS-CoV-2G or pCMV-VSV-G) according to manufacturer’s instructions. Pseudotyped virions were efficiently released in the supernatant and collected for the use in experiments.

### Construction of AAV vectors expressing hACE2 and production of pseudotyped AAV

The gene encoding human angiotensin-converting enzyme 2 (hACE2, NCBI Reference Sequence: NM_021804.3) were PCR-amplified using phACE2-F (5’-GGAATTGTACCCGCGGCCGCCATGTCAAGCTCTTCCTGGCT-3’) and phACE2-R (5’-GATAAGCTTGATGCGGCCGCCTAAAAGGAGGTCTGAACATCATCAGTG-3’) primers and cloned into a NotI-linearized ssAAV vector [78] using the In-Fusion HD Cloning Plus kit (Takara Bio, Shiga, Japan). For analyzing the tissue distribution of exogenous protein expression, double stranded AAV vectors containing the green fluorescent protein (GFP) gene driven by CB promoter [79] were used. The recombinant AAV vectors were produced by a triple transfection method as previously described [80] and purified by cesium chloride sedimentation. The physical vector titers were assessed by real-time PCR using SYBR Green reaction mix (Roche Diagnostics, Mannheim, Germany) [81].

### Western blot analysis of hACE2 expression

3T3 cells were transiently transfected with AAV-CB-hACE2 plasmid by calcium phosphate transfection and lysed with RIPA buffer (50 mM Tris-HCl, pH 7.4, 150 mM NaCl, 2 mM EDTA, 1% NP-40, 0.5% Sodium deoxycholate, 0.1% Sodium dodecyl sulfate, 50 mM Sodium fluoride) containing complete protease inhibitor cocktail (Roche Diagnostics, Mannheim, Germany) at 72 hours post transfection. The cell lysate was then separated by 4–12% Bis-Tris gradient gel (NuPAGE, Thermo Scientific, IL, USA) and transferred to the PVDF membranes (Bio-Rad, CA, USA). The transferred membranes were blocked with 5% BSA in PBST (PBS containing 0.05% Tween-20) for an hour at room temperature and then incubated with either mouse anti-hACE2 (R&D, MN, USA) or mouse anti-actin antibody (Sigma-Aldrich, MO, USA) in the blocking buffer overnight at 4°C. HRP-conjugated goat-anti-mouse IgG Fc (Chemicon, CA, USA) was served as the secondary antibody. The blots were visualized with ECL western blot substrate (Millipore, MA, USA) and analyze by LAS 3000 (Fujifilm, Tokyo, Japan).

### In vitro binding assay

To examine hACE2 expression, 3T3 cells were transduced by AAV6/hACE2 or AAV9/hACE2 for 48 hours and dissociated by Versene solution (Thermo Fisher Scientific, MA, USA) at 37°C for 30 minutes. For each test, 2×10^5^ cells were resuspended in 100 μl of Dulbecco’s phosphate buffered saline (DPBS) containing 1% FBS and then incubated with 0.2 μg of recombinant SARS-CoV-2 Spike protein (Sino Biological, Beijing, China) for 1 hour on ice. The recombinant protein consists of the receptor binding domain (RBD) of SARS-CoV-2 Spike protein and a mouse Fc region at the C-terminus. Next, the cells were incubated with 100 μl PE-labeled goat anti-mouse IgG-Fc antibody (Jackson Immuno Research, PA, USA) for 30 minutes. After the staining procedure, cells were resuspended again in 300 μl of 1% FBS-DPBS containing 0.25 μg of 7-amino-actinomycin D (Thermo Fisher Scientific, CA, USA) to exclude non-viable cells, and then analyzed by LSRII flow cytometer (BD Biosciences, MA, USA).

### AAV infection in mice

AAV6/hACE2 and AAV9/hACE2 were produced by AAV core facility in Academia Sinica. Eight to ten-week-old C57BL/6 mice were anaesthetized by intraperitoneal (i.p.) injection of a mixture of Atropine (0.4 mg/ml, Astar Pharmaceutical Co., Hsinchu, Taiwan)/Ketamine (20 mg/ml, Ketalar, Pfizer, PA, USA)/Xylazine (0.4%, Rompun, Bayer, PA, USA). Mice were then intratracheally or intranasally injected with 3 x 10^11^ vg of AAV6/hACE2 in 50 μl saline as previously described [42]. To transduce extrapulmonary organs, 1 x 10^12^ vg of AAV9/hACE2 [82] in 100 ul saline were i.p. injected into mice.

### GFP signal analysis

Three weeks after the injection of AAV6/EGFP (3x10^11^ vg/mouse) or AAV9/EGFP (1x10^12^ vg/mouse), the mice were anesthetized and perfused transcardially with ice-cold phosphate-buffered saline (PBS) followed by 4% paraformaldehyde (PFA, Sigma-Aldrich, CA,USA). Various tissues were collected and post-fixed with 4% PFA overnight. Subsequently, tissues were dehydrated in 15% and 30% sucrose until they sank, and then embedded in Tissue-Tek O.C.T. Compound (Sakura Finetek, CA, USA). 10-μm-thick tissue sections were stained with 300 μM DAPI (Thermo Fisher Scientific, CA, USA) for 10 minutes. GFP and DAPI signals were visualized under a laser scanning confocal microscope LSM700 (Carl Zeiss Microscopy GmbH, Jena, Germany).

### SARS-CoV-2 challenge

Two weeks after AAV6/hACE2 and AAV9/hACE2 transduction, mice were anesthetized and intranasally (i.n.) challenged with 1x10^5^ TCID50 of SARS-CoV-2 in a volume of 100 μl. K18-hACE2 mice were also challenged with the same SARS-CoV-2 dose under anesthetization. For examining the sustainability of hACE2 expression (Fig 2A), naïve AAV/hACE2 mice were maintained up to 28 weeks and, and euthanized at 1, 2, 3, 4, 8, 20 and 28 week post infection. To make the long-term observation of mice weight upon SARS-CoV-2 challenge (Fig 3B), both naïve and infected AAV/hACE2 mice were kept for 14 days and then euthanized. For other experiments, mice were sacrificed for the sample collection at the indicated time points. All mouse challenge experiments were evaluated and approved by the IACUC of Academia Sinica and conducted in the animal biological safety level 3 (ABSL-3) facility at Genomics Research Center, Academia Sinica.

### Immunohistochemistry and immunofluorescence staining

Mice organs were collected after the euthanasia with an avertin overdose (i.p.). For each mouse, left lobe of the lung and a half portion of the heart were fixed in 4% PFA for 72 hours. Subsequently, fixed organs were processed for paraffin embedding and then cut into 5-μm-thick sections for immunohistochemistry. Theses tissue sections were deparaffined in xylene and rehydrated through a graded series of ethanol baths. The sections were treated with 3% H_2_O_2_ for 30 minutes at room temperature (RT) to quench endogenous peroxidase, washed three times with PBS, and then heated in sodium citrate buffer (pH 6.0) for 30 minutes. After cooling, the sections were washed with PBS for 5 minutes twice. Non-specific antibody binding was blocked by 3% BSA in PBS for 30 minutes at RT. The staining with the primary polyclonal anti-hACE2 antibody (Abcam, Inc., MA, USA) or anti-SARS-CoV-2 N protein antibody (a gift kindly provided by Dr. An-Suei Yang, Genomics Research Center, Academia Sinica) was conducted overnight at 4°C, followed by 3 times of washing with PBS for 5 minutes. Subsequently, secondary alkaline phosphatase (AP)-conjugated anti-rabbit IgG antibody (Jackson ImmunoResearch, PA, USA) for hACE2 staining or horseradish peroxidase (HRP)-conjugated goat anti-human IgG secondary antibody (A0170; Sigma-Aldrich, MO, USA) for N protein was applied to the samples for 30 minutes at RT. After washing with PBS for 5 minutes three times, the color reaction of stained samples was initiated by adding AP substrate (Vector Labs, CA, USA) or AEC substrate (DAKO, CA, USA) onto the slides for 3 minutes and stopped by washing in PBS. All slides were counterstained with hematoxylin. Images of the tissues were acquired using an Olympus BX51 microscope equipped with a digital camera (Olympus Corporation, Tokyo, Japan).

For immunofluorescence microscopy, tissue sections were stained with indicated primary antibody and secondary Alexa Fluor 488-labeled goat anti-human ΙgG (Invitrogen, OR, USA), each for 1 h at room temperature. Nuclei were counterstained with 300 μM DAPI (Invitrogen, Paisley, UK). The slides were imaging using the confocal microscope LSM700 Stage (Carl Zeiss Microscopy GmbH, Jena, Germany).

### Histopathological examination of the tissue

As described in the “Immunohistochemistry” session, tissues were collected and fixed in 4% PFA. After one week of fixation, the tissues were processes for paraffin embedding, sectioning, and Hematoxylin and Eosin (H&E) staining. Tissue sections were examined by microscopy. The pathophysiology of infected mice lungs was evaluated according to a lung histopathological scoring system as previously described [24,48].

### RNA extraction and RT-QPCR for hACE2 and SARS-CoV-2 RNA quantification

Mouse tissues were weighted and homogenized using the SpeedMill PLUS (Analytik Jena AG, Jena, Germany) for 2 rounds, each round for 2 minutes in the lysis buffer. Homogenates were centrifuged at 3,000 rpm for 5 minutes at 4°C. The supernatant was collected, and total RNA was then extracted by using RNeasy Mini Kit (QIAGEN, Hilden, Germany) following the manufacturer’s instructions. For each sample, a total of 30 μl total RNA solution was obtained.

For measuring hACE2 expression, total RNA was subjected to RT-QPCR using the primer/probe set for hACE2 as previously described [43]; the results were compared with an DNA standard curve and normalized to ng of total RNA to obtain the copy number of hACE2 RNA.

To measure the level of SARS-CoV-2 genomic RNA, specific primers targeting 26,141 to 26,253 region in the envelope (E) gene of SARS-CoV-2 genome were used for TaqMan real-time RT-QPCR method described in the previous study [83]. Forward primer E-Sarbeco-F1 (5’-ACAGGTACGTTAATAGTTAATAGCGT-3’) and the reverse primer E-Sarbeco-R2 (5’-ATATTGCAGCAGTACGCACACA-3’), in addition to the probe E-Sarbeco-P1 (5’-FAM-ACACTAGCCATCCTTACTGCGCTTCG-BBQ-3’), were used in the assay. Additionally, negative-stranded vRNA was also quantified to assess active virus replication as described previously [62]. In brief, a negative-RNA specific primer targeting E gene was added in the reverse transcription (RT) step to generate cDNA. After the heat-inactivation, RT-QPCR was then performed with identical primers and the aforementioned probe for E gene (E-Sarbeco-F1/R2/P1).

RT-QPCR of subgenomic (sg) viral RNA of E gene was modified from E RT-QPCR by replacing the forward primer with sgLeadSARSCoV2-F (5’-CGATCTCTTGTAGATCTGTTCTC-3’), which specifically targeted the leader sequence of subgenomic RNA.

The RT-QPCR was performed using Superscript III one-step RT-QPCR system with Platinum Taq Polymerase (Thermo Fisher Scientific, CA, USA). For each reaction, 5 μl of RNA sample was added into a total of 25 μl solution containing 400 nM forward and reverse primers, 200 nM probe, 1.6 mM deoxy-ribonucleoside triphosphate (dNTP), 4 mM magnesium sulphate, 50 nM ROX reference dye, and 1 μl of enzyme mixture from the kit. The RT-QPCR reaction was performed following a one-step PCR protocol: 1) 10 minutes at 55°C for cDNA synthesis, 2) 3 minutes at 94°C, and 3) 45 amplification cycles of 94°C for 15 seconds and 58°C for 30 seconds. The reaction and analysis were carried out by Applied Biosystems 7500 Real-Time PCR System (Thermo Fisher Scientific, CA, USA). The synthetic 113-bp (E) and 171-bp (sgE) oligonucleotide fragments were used as the QPCR standard to estimate copy numbers of viral genome or subgenomic RNA. The oligonucleotide was synthesized by Genomics BioSci and Tech Co. Ltd. (Taipei, Taiwan).

### Quantification of viral titer by the tissue culture infectious dose assay

Mouse tissues were homogenized in 1 ml of DMEM supplemented with 2% FBS and 1% P/S using a homogenizer. After centrifugation at 13,000 rpm for 10 minutes, the supernatant was collected for virus titration. Briefly, 10-fold serial dilutions of each sample were added onto Vero-E6 cell monolayer in quadruplicate, and cells were incubated for 4 days for the observation of cytopathic effects. The fifty-percent tissue culture infectious dose (TCID_50_) per milliliter was calculated by the Reed and Muench method.

### Preparation of blood samples

Mice whole blood samples were collected into BD Microtainer K2EDTA tubes (BD Biosciences, NJ, USA) by cardiac puncture at the indicated time points. The blood samples were fractionated by centrifugation at 400 x g for 5 minutes at RT, and the plasma was collected and stored at -80°C for cytokine and chemokine multiplex assay. Red blood cells were lysed twice with ammonium-chloride-potassium (ACK) lysing buffer (Gibco, Thermo Fisher Scientific, CA, USA) for 2–3 minutes at RT before being washed twice with DPBS containing 2% FBS. The remaining cells were resuspended in DPBS/2% FBS on ice for FACS analysis.

### Fluorescence-activated cell sorting (FACS) analysis of peripheral leukocyte populations

To analyze the cell composition in the circulatory system by flow cytometry, peripheral blood mononuclear cells (PBMCs) were washed with PBS, blocked with a homemade anti-mouse CD16/32 antibody (2.4G2), and then stained with antibodies against various cell markers in FACS buffer (PBS supplemented with 1% FBS). The staining reagents included APC anti-CD45 (30-F11), FITC anti-CD11b (M1/70), PerC:P-Cy5.5 anti-Ly6G (1A8), PE anti-B220 (RA3-6B2), and Brilliant violet 421 anti-TCRb (H57-597) antibodies from BD Biosciences (NJ, USA), as well as APC-Cy7 anti-Ly6C (HK1.4) antibody from BioLegend (CA, USA). The staining for surface antigens was performed on ice for 30 minutes. All cytometric data were collected on FACSCelesta (BD Biosciences, CA, USA) and analyzed with the FlowJo software (Tree Star, Inc., OR, USA). Dead cells and non-singlet events were excluded from analyses based on the staining of Fixable viability dye eFluor 506 (eBioscience, CA, USA) and characteristics of forward- and side-scattering.

### Multiplex assay for cytokine and chemokine proteins

Concentrations of a broad panel of cytokines and chemokines in plasma (CCL3, CCL4, CCL2, CCL7, CCL11, CXCL1, CXCL2, CXCL10, IL-1β, IL-2, IL-4, IL-5, IL-6, IL-9, IL-10, IL-12p70, IL-13, IL17A, IL-23, IL-27, IL-18, IL-22, IL-23, TFN-α, IFN-γ, GM-CSF) were determined by using the ProcartaPlex Mouse Cytokine and Chemokine Panel 1 (26-plex) kit (Thermo Fisher Scientific, MA, USA), following the manufacturer’s instructions. Samples were measured on a Luminex 200 instrument (Luminex, Texas, USA) and the data analysis was conducted by using ProcartaPlex Analyst 1.0 software (Thermo Fisher Scientific, MA, USA).

### Efficacy test of the neutralizing antibody cocktail

Two weeks after AAV/hACE2 transduction, groups of mice (n = 4 for each group) were challenged with 1 x 10^5^ TCID_50_ of SARS-CoV-2 via i.n. injection. On day 1 post-infection, mice were intraperitoneally treated with a cocktail of RBD-chAb-28 antibody and RBD-chAb-51 antibody (10 mg/kg each) or a control isotype monoclonal antibody (20 mg/kg). The viral infection in mice lungs was examined on day 5 post-infection.

### Graphical illustrations

Graphical illustrations were created using Biorender.com. Data visualization was performed by GraphPad Prism version 7 (GraphPad Software Inc., CA, USA).

### Statistical analysis

Results are presented as the mean ± standard deviation (SD). Differences between experimental groups of animals were analyzed by Student’s t test; p < 0.05 was considered as statistically significant.

## Supporting information

S1 Fig Exogenous gene expression in mouse tissues by recombinant AAV/EGFP transductions. C57BL/6 mice were transduced with recombinant AAV vectors carrying EGFP via different administration routes. AAV6/EGFP was administrated intratracheally or intranasally, and AAV9/EGFP was administrated intraperitoneally. Tissue cryo-sections were prepared 3 weeks after AAV administration (n = 2–3 for each group), and images were acquired by confocal microscopy (blue, DAPI; green: EGFP). WT, non-transduced wild-type mice. Scale bar, 100 μm.
(TIF)

S2 Fig hACE2 expression in AAV/hACE2 mice and K18-hACE2 mice. (A) RT-QPCR analysis of hACE2 expression level in the liver of non-transduced wild type (WT, n = 3), AAV/empty-transduced (n = 3), and AAV/hACE2-transduced (n = 3 or 4) mice. (B) Representative immunohistochemical staining of hACE2 in the liver of control (non-transduced) and AAV/hACE2-transduced mice. Tissues were analyzed at week 3 post AAV/hACE2 administration. Scale bar, 100 μm. (C) RT-QPCR analysis of hACE2 expression level in the lung of AAV/hACE2 mice (n = 4) and K18-hACE2 transgenic mice (n = 3). (C) RT-QPCR analysis of hACE2 expression level in the lung of WT (n = 3) and AAV/hACE2 mice at different time points (n = 2 at week 20; = 3 at week 28). P values were calculated by two-tailed unpaired Student’s t test (*, P < 0.05; **, P < 0.005; ***, P < 0.0005).
(TIF)

S3 Fig Infection of SARS-CoV-2 pseudoviruses in AAV/hACE2 mice. Non-transduced wild-type (WT) mice were subjected to mock or VSV-G pseudotyped virus infection. AAV/hACE2 mice were infected with VSV-based pseudotyped SARS-CoV-2. Both pseudotyped viruses carried RFP as the reporter. Images of tissue cryosections were acquired by fluorescent microscopy (blue, DAPI; red, RFP). Scale bar, 100 μm. N = 2–3 for each group of mice.
(TIF)

S4 Fig The body weight changes in SARS-CoV-2-infected AAV/hACE2 mice transduced via i.t. administration alone. The weight changes of non-transduced wild-type (WT, black circle) and AAV/hACE2 (red triangle) mice challenged with SARS-CoV-2 at different days post-infection (dpi) (n = 3 for each condition).
(TIF)

S5 Fig Establishment of SARS-CoV-2 infection in K18-hACE2 mice and AAV/hACE2 BALB/c mice. K18-hACE2 transgenic (n = 3), non-transduced wild-type (WT) mice (n = 4), and AAV/hACE2-transduced BALB/c or B6 mice (n = 4 for each type of mice) were challenged with SARS-CoV-2. All mice were sacrificed at 5 days post-infection, and the lungs were collected for further analyses. (A and B) The numbers of viral genomic RNA (A) and infectious virion (B) in the lungs of K18-hACE2 and AAV/hACE2-B6 mice were measured by RT-QPCR and TCID_50_ analysis, respectively. (C and D) The numbers of viral genomic RNA (C) and infectious virion (D) in the lungs of WT and AAV/hACE2-BALB/c mice were measured by RT-QPCR and TCID_50_ analysis, respectively. (E) Histopathological scores of the lung pathology in the WT and AAV/hACE2-BALB/c were analyzed by H&E examination.
(TIF)

S6 Fig Detection of SARS-CoV-2 N protein in the lungs of infected mice. Non-transduced wild-type (WT) and AAV/hACE2 mice (n = 4 for each group) were challenged with SARS-CoV-2 and sacrificed at 5 days post-infection for examining the infection by immunofluorescence (A) or immunohistochemical (B) staining of viral N protein. (A) For the immunofluorescence staining, the whole lung images were acquired by digital slide scanner (blue, DAPI; green, SARS-CoV-2 N protein). Scale bar, 1 mm. (B) The samples were also subjected to hemotoxylin counterstain. Scale bar, 100 μm.
(TIF)

S7 Fig Histologic examination of the heart collected from SARS-CoV-2-infected mice. Non-transduced wild-type (WT) and AAV/hACE2 mice (n = 4 for each group) were challenged with SARS-CoV-2 and sacrificed at 5 days post-infection for H&E stain and histologic examination. Vacuolar degeneration was observed in the heart (arrow, inset). Scale bars, 100 μm.
(TIF)

S8 Fig Establishment of SARS-CoV-2 infection and pathology in the liver of AAV/hACE2 mice. Non-transduced wild-type (WT) and AAV/hACE2 mice (n = 3~4 for each group) were challenged with SARS-CoV-2 and sacrificed for further examination. (A) The numbers of viral genomic RNA in the liver of WT (black circle) and AAV/hACE2 (red triangle) mice at 2, 5, 7, and 14 dpi. Dashed lines indicate the limit of detection (LOD, 10^2^ viral genomes/μg of total RNA). Bars indicate mean values. P values were calculated by two-tailed unpaired Student’s t test (*, P < 0.05). (B) H&E stain of the liver of WT and AAV/hACE2 mice on day 5 post infection. Scale bar, 100 μm.
(TIF)

S9 Fig Plasma chemokine/cytokine profiles of SARS-CoV-2-infected mice. Plasma chemokine/cytokine levels in non-transduced wild-type (WT) and AAV/hACE2 mice infected with SARS-CoV-2 were measured by multiplex assay at 0, 2, 5, 7 days post-infection (dpi) (WT: n = 3; AAV/hACE2: n = 3 for 0, 2 and 7 dpi, n = 4 for 5 dpi)
(TIF)

S10 Fig Quantification of subgenomic viral E RNA in the lung of infected AAV/hACE2 mice upon RBD-chAbs cocktail treatment. The numbers of subgenomic (sg) viral E RNA (A) and negative-strand E RNA (B) in the lungs of AAV/hACE2 mice treated with isotype control (black circle, n = 4) or RBD-chAbs cocktail (red triangle, n = 4) were measured by RT-QPCR. The dashed line indicates the limit of detection. Bars indicate mean values. P values were calculated by two-tailed unpaired Student’s t test (*, P < 0.05).
(TIF)

S11 Fig hACE2 expression and establishment of SARS-CoV-2 infection in AAV/hACE2 mice. Non-transduced wild-type (WT) mice, and AAV/hACE2 mice transduced with different conditions were challenged with SARS-CoV-2 (n = 3 for each type of mice). All mice were sacrificed at 5 days post-infection, and the organs were collected for further analyses. (A) The expression level of hACE2 and the numbers of viral genomic RNA in the brain of AAV/hACE2 mice transduced via i.t. and i.p. routes were measured by RT-QPCR. (B) The expression level of hACE2 and the numbers of viral genomic RNA in the nasal turbinate of AAV/hACE2 mice transduced via i.t, i.p., and i.n. routes were measured by RT-QPCR. The dashed line indicates the limit of detection. Bars indicate mean values. P values were calculated by two-tailed unpaired Student’s t test (*, P < 0.05; **, P<0.005).
(TIF)
