# Supplementary figures and images for: Rapid generation of mouse model for emerging infectious disease with the case of severe COVID-19
Source: PLoS Pathog. 2021 Aug 11;17(8):e1009758. doi: 10.1371/journal.ppat.1009758 (PMC8415591; doi:10.1371/journal.ppat.1009758)

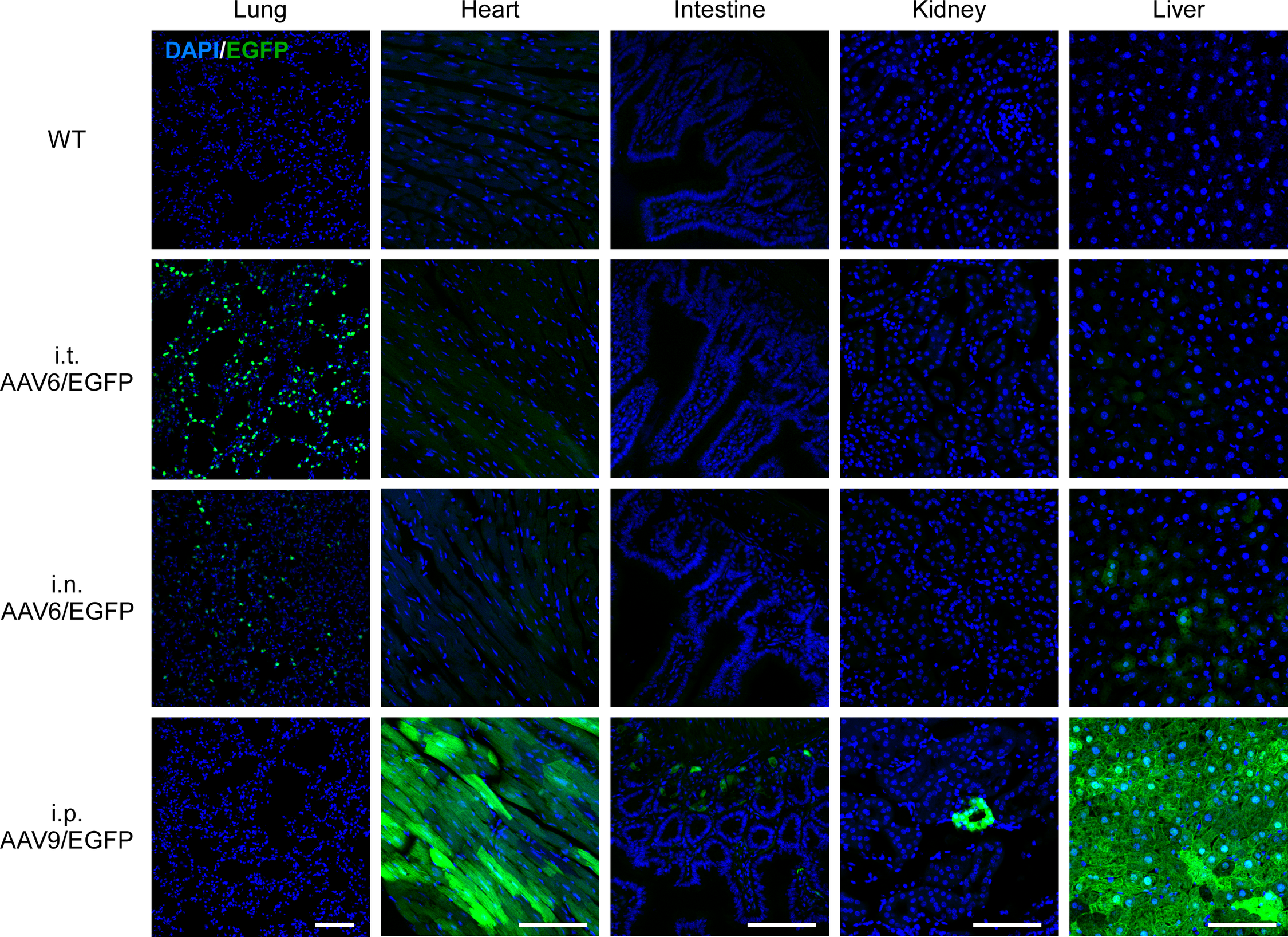

Supplement: S1 Fig — C57BL/6 mice were transduced with recombinant AAV vectors carrying EGFP via different administration routes. AAV6/EGFP was administrated intratracheally or intranasally, and AAV9/EGFP was administrated intraperitoneally. Tissue cryo-sections were prepared 3 weeks after AAV administration (n = 2–3 for each group), and images were acquired by confocal microscopy (blue, DAPI; green: EGFP). WT, non-transduced wild-type mice. Scale bar, 100 μm. (TIF) [file ppat.1009758.s001.tif]

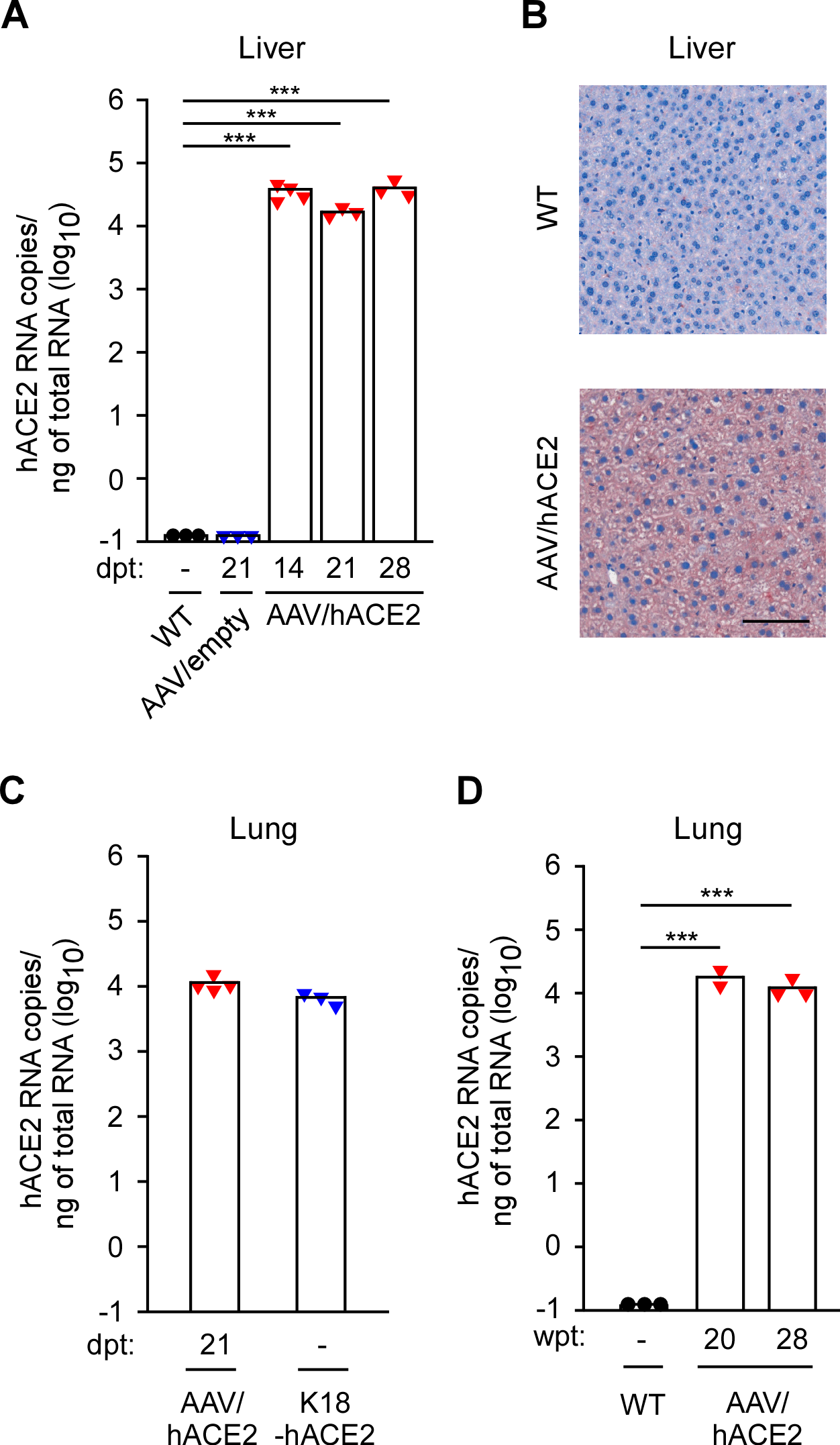

Supplement: S2 Fig — (A) RT-QPCR analysis of hACE2 expression level in the liver of non-transduced wild type (WT, n = 3), AAV/empty-transduced (n = 3), and AAV/hACE2-transduced (n = 3 or 4) mice. (B) Representative immunohistochemical staining of hACE2 in the liver of control (non-transduced) and AAV/hACE2-transduced mice. Tissues were analyzed at week 3 post AAV/hACE2 administration. Scale bar, 100 μm. (C) RT-QPCR analysis of hACE2 expression level in the lung of AAV/hACE2 mice (n = 4) and K18-hACE2 transgenic mice (n = 3). (C) RT-QPCR analysis of hACE2 expression level in the lung of WT (n = 3) and AAV/hACE2 mice at different time points (n = 2 at week 20; = 3 at week 28). P values were calculated by two-tailed unpaired Student’s t test (*, P < 0.05; **, P < 0.005; ***, P < 0.0005). (TIF) [file ppat.1009758.s002.tif]

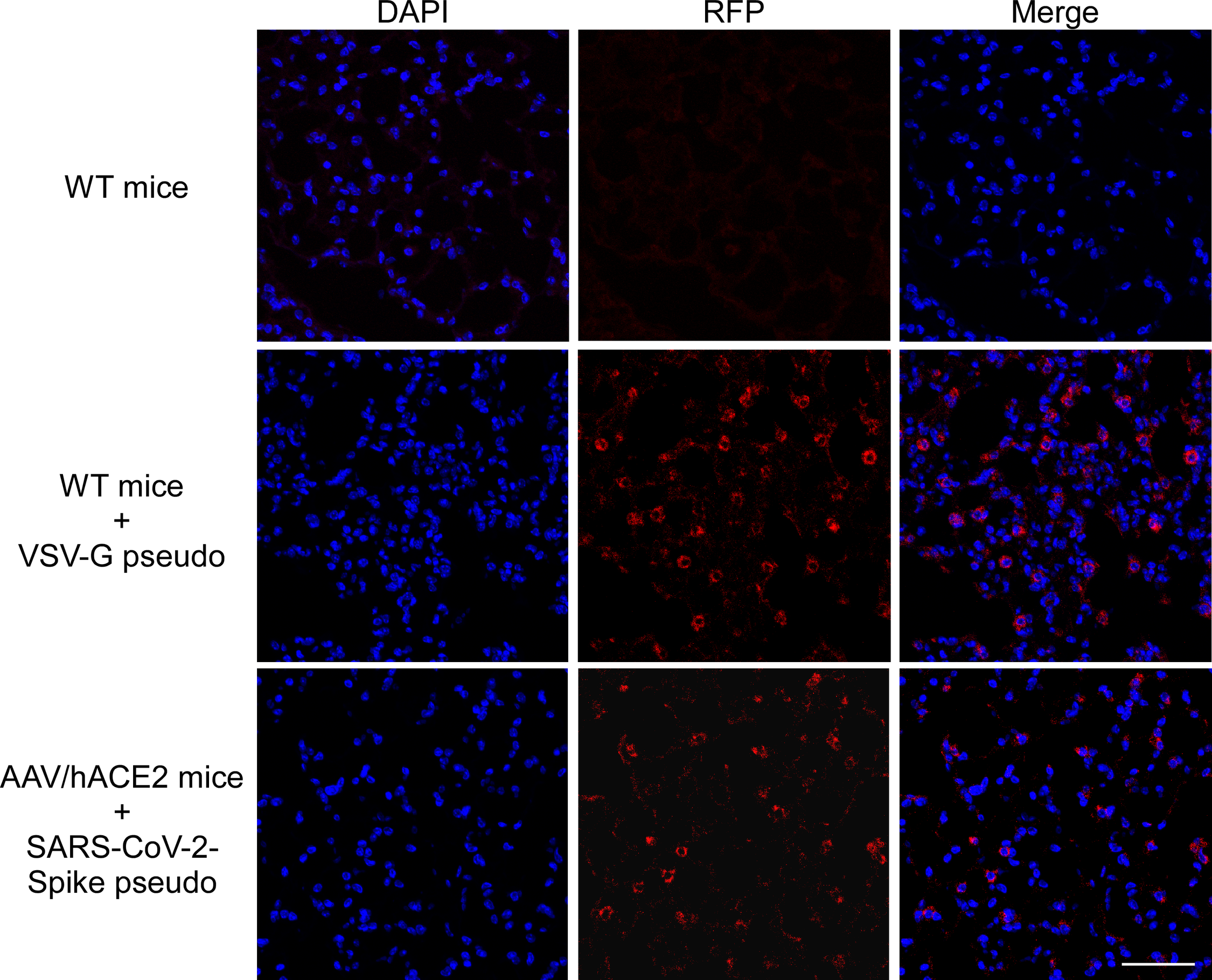

Supplement: S3 Fig — Non-transduced wild-type (WT) mice were subjected to mock or VSV-G pseudotyped virus infection. AAV/hACE2 mice were infected with VSV-based pseudotyped SARS-CoV-2. Both pseudotyped viruses carried RFP as the reporter. Images of tissue cryosections were acquired by fluorescent microscopy (blue, DAPI; red, RFP). Scale bar, 100 μm. N = 2–3 for each group of mice. (TIF) [file ppat.1009758.s003.tif]

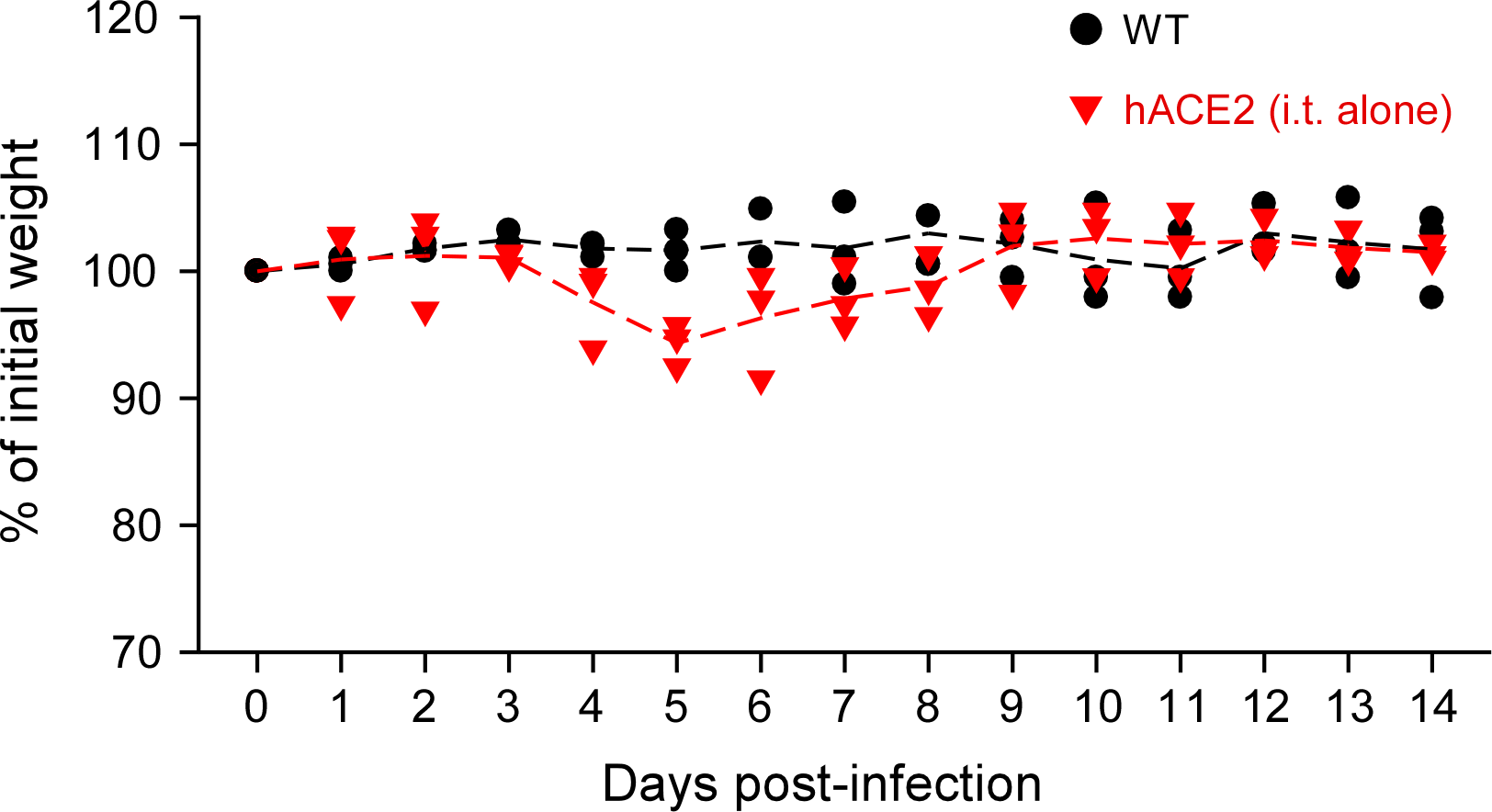

Supplement: S4 Fig — The weight changes of non-transduced wild-type (WT, black circle) and AAV/hACE2 (red triangle) mice challenged with SARS-CoV-2 at different days post-infection (dpi) (n = 3 for each condition). (TIF) [file ppat.1009758.s004.tif]

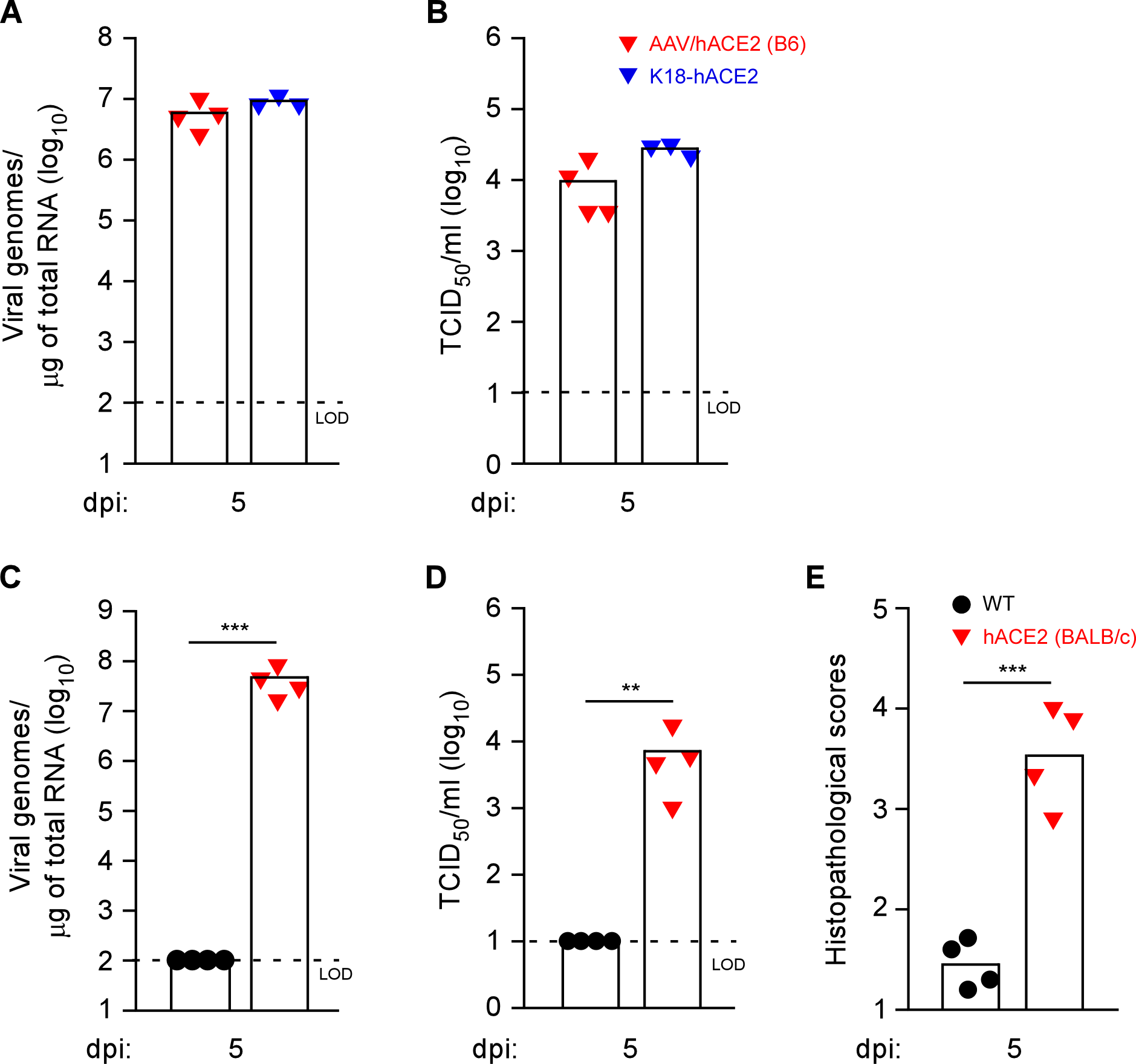

Supplement: S5 Fig — K18-hACE2 transgenic (n = 3), non-transduced wild-type (WT) mice (n = 4), and AAV/hACE2-transduced BALB/c or B6 mice (n = 4 for each type of mice) were challenged with SARS-CoV-2. All mice were sacrificed at 5 days post-infection, and the lungs were collected for further analyses. (A and B) The numbers of viral genomic RNA (A) and infectious virion (B) in the lungs of K18-hACE2 and AAV/hACE2-B6 mice were measured by RT-QPCR and TCID50 analysis, respectively. (C and D) The numbers of viral genomic RNA (C) and infectious virion (D) in the lungs of WT and AAV/hACE2-BALB/c mice were measured by RT-QPCR and TCID50 analysis, respectively. (E) Histopathological scores of the lung pathology in the WT and AAV/hACE2-BALB/c were analyzed by H&E examination. (TIF) [file ppat.1009758.s005.tif]

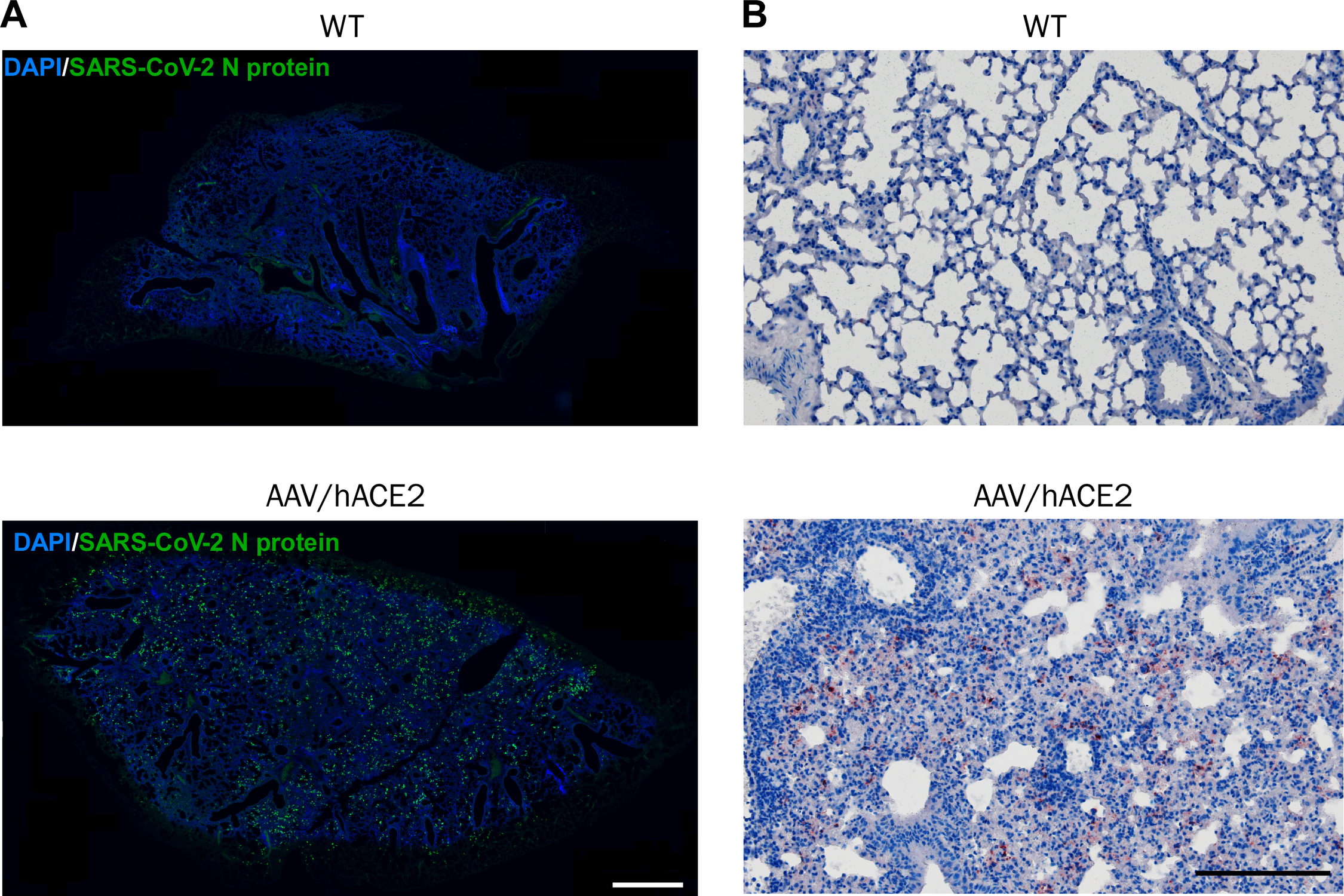

Supplement: S6 Fig — Non-transduced wild-type (WT) and AAV/hACE2 mice (n = 4 for each group) were challenged with SARS-CoV-2 and sacrificed at 5 days post-infection for examining the infection by immunofluorescence (A) or immunohistochemical (B) staining of viral N protein. (A) For the immunofluorescence staining, the whole lung images were acquired by digital slide scanner (blue, DAPI; green, SARS-CoV-2 N protein). Scale bar, 1 mm. (B) The samples were also subjected to hemotoxylin counterstain. Scale bar, 100 μm. (TIF) [file ppat.1009758.s006.tif]

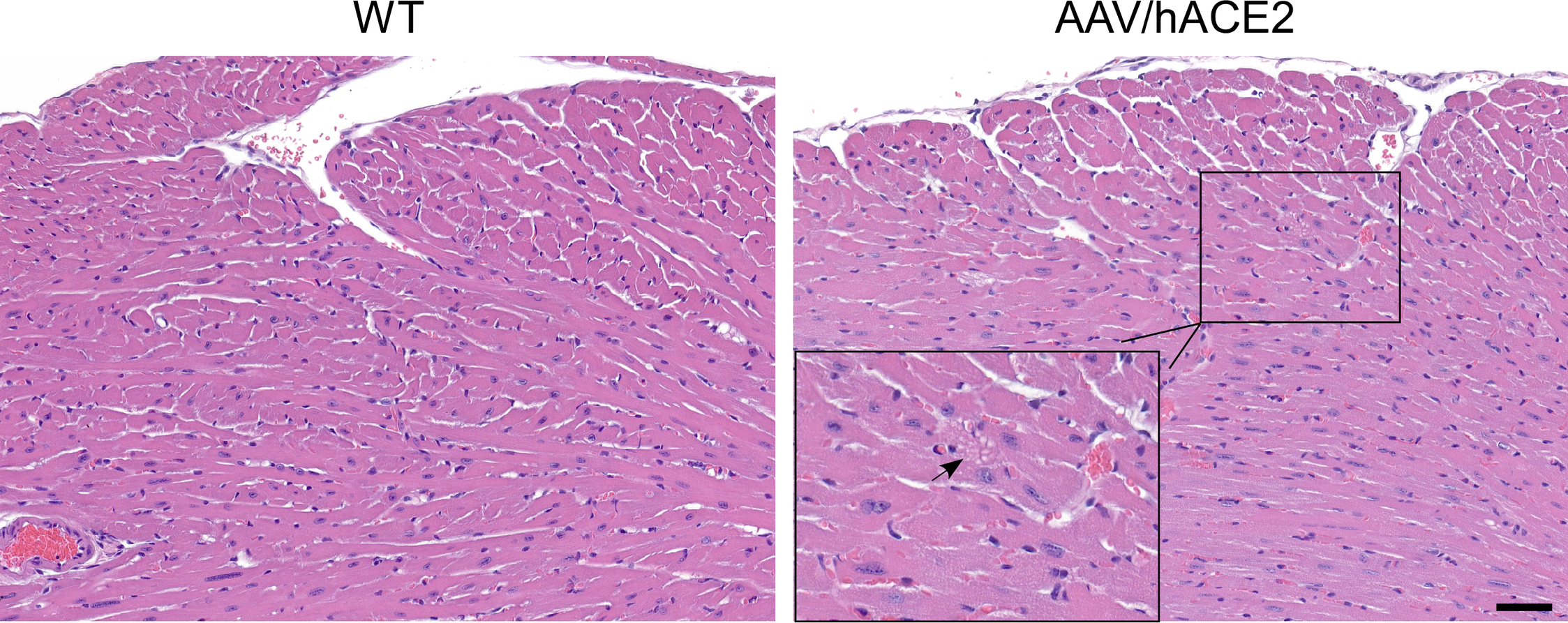

Supplement: S7 Fig — Non-transduced wild-type (WT) and AAV/hACE2 mice (n = 4 for each group) were challenged with SARS-CoV-2 and sacrificed at 5 days post-infection for H&E stain and histologic examination. Vacuolar degeneration was observed in the heart (arrow, inset). Scale bars, 100 μm. (TIF) [file ppat.1009758.s007.tif]

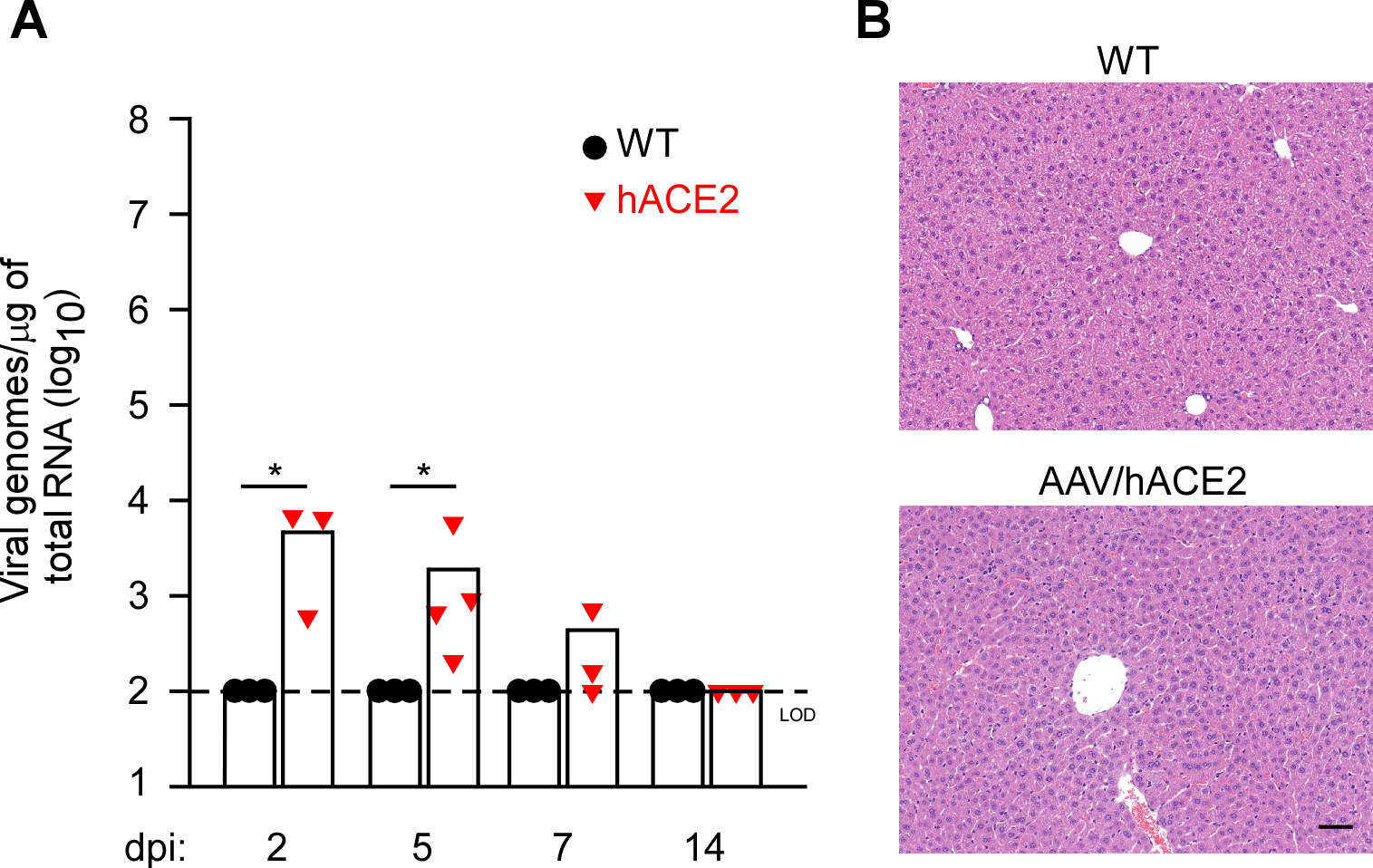

Supplement: S8 Fig — Non-transduced wild-type (WT) and AAV/hACE2 mice (n = 3~4 for each group) were challenged with SARS-CoV-2 and sacrificed for further examination. (A) The numbers of viral genomic RNA in the liver of WT (black circle) and AAV/hACE2 (red triangle) mice at 2, 5, 7, and 14 dpi. Dashed lines indicate the limit of detection (LOD, 102 viral genomes/μg of total RNA). Bars indicate mean values. P values were calculated by two-tailed unpaired Student’s t test (*, P < 0.05). (B) H&E stain of the liver of WT and AAV/hACE2 mice on day 5 post infection. Scale bar, 100 μm. (TIF) [file ppat.1009758.s008.tif]

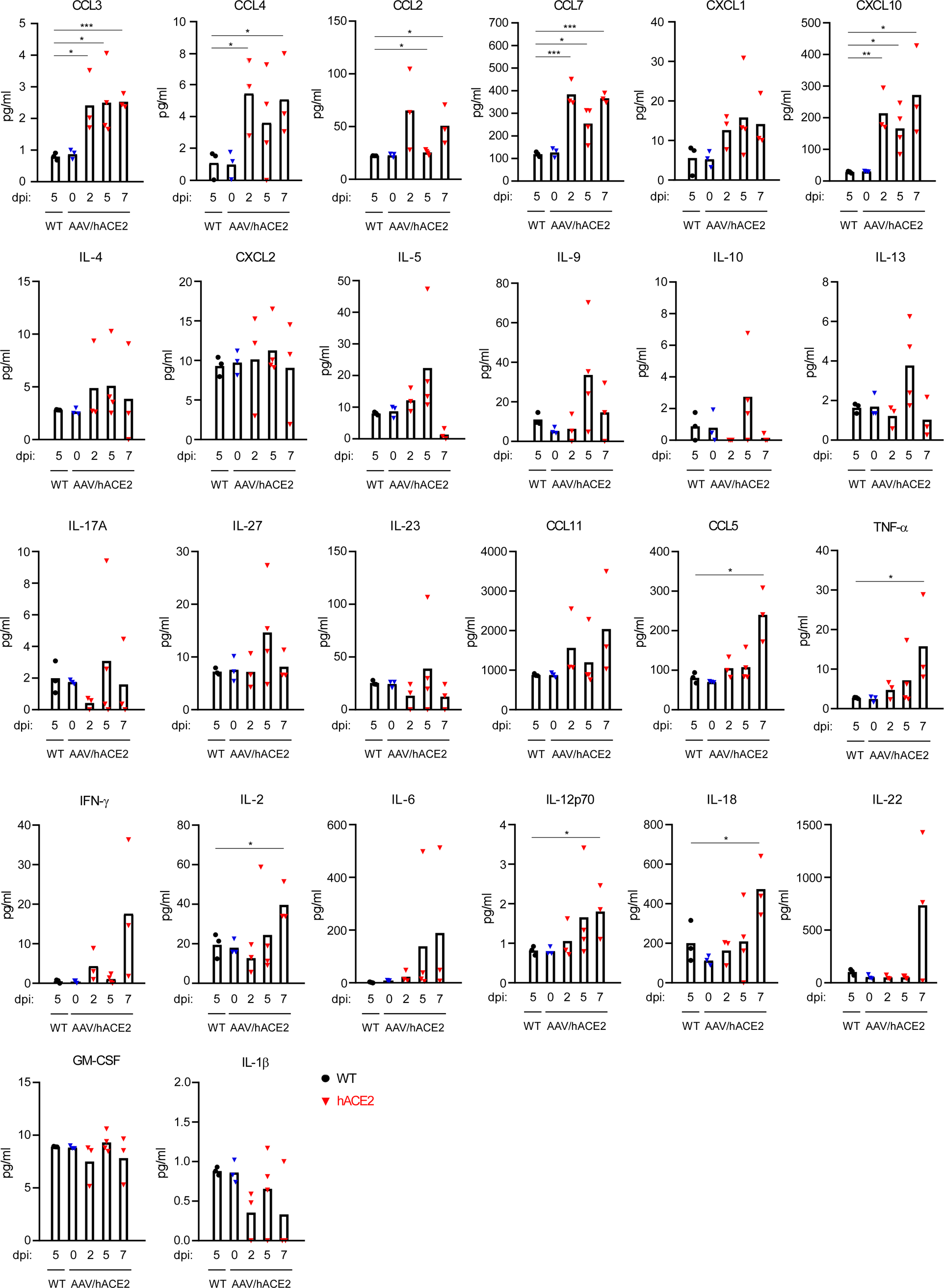

Supplement: S9 Fig — Plasma chemokine/cytokine levels in non-transduced wild-type (WT) and AAV/hACE2 mice infected with SARS-CoV-2 were measured by multiplex assay at 0, 2, 5, 7 days post-infection (dpi) (WT: n = 3; AAV/hACE2: n = 3 for 0, 2 and 7 dpi, n = 4 for 5 dpi) (TIF) [file ppat.1009758.s009.tif]

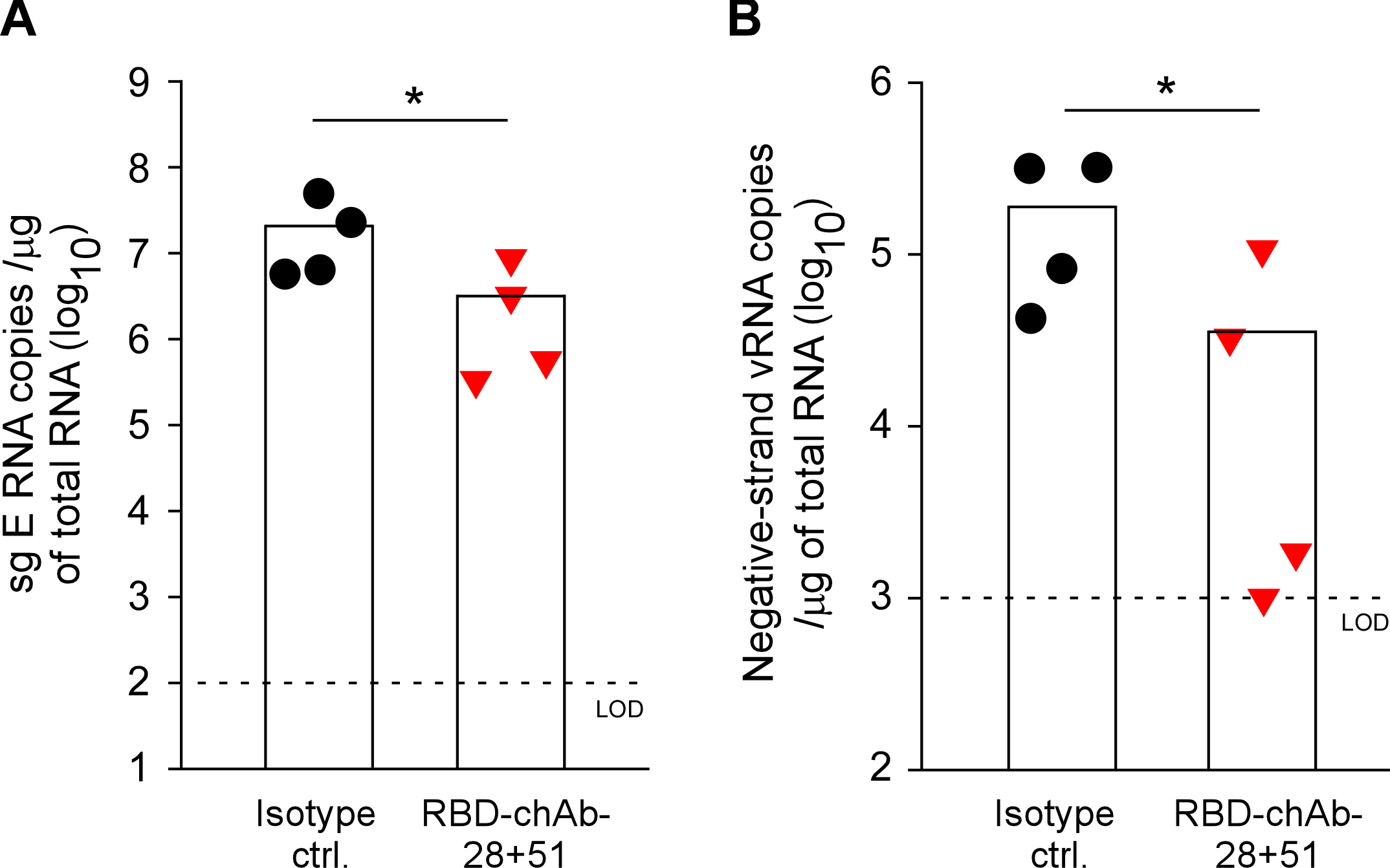

Supplement: S10 Fig — The numbers of subgenomic (sg) viral E RNA (A) and negative-strand E RNA (B) in the lungs of AAV/hACE2 mice treated with isotype control (black circle, n = 4) or RBD-chAbs cocktail (red triangle, n = 4) were measured by RT-QPCR. The dashed line indicates the limit of detection. Bars indicate mean values. P values were calculated by two-tailed unpaired Student’s t test (*, P < 0.05). (TIF) [file ppat.1009758.s010.tif]

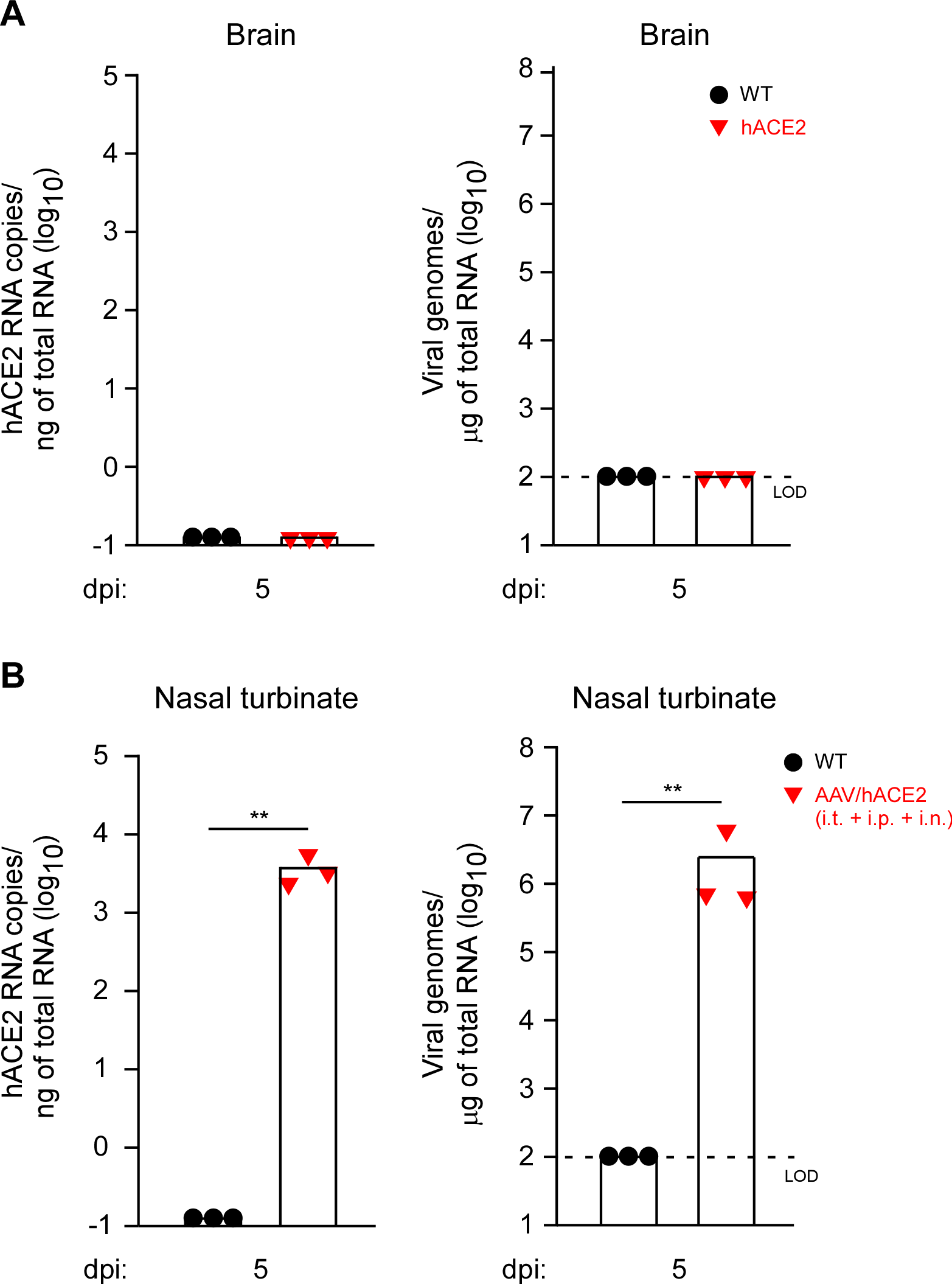

Supplement: S11 Fig — Non-transduced wild-type (WT) mice, and AAV/hACE2 mice transduced with different conditions were challenged with SARS-CoV-2 (n = 3 for each type of mice). All mice were sacrificed at 5 days post-infection, and the organs were collected for further analyses. (A) The expression level of hACE2 and the numbers of viral genomic RNA in the brain of AAV/hACE2 mice transduced via i.t. and i.p. routes were measured by RT-QPCR. (B) The expression level of hACE2 and the numbers of viral genomic RNA in the nasal turbinate of AAV/hACE2 mice transduced via i.t, i.p., and i.n. routes were measured by RT-QPCR. The dashed line indicates the limit of detection. Bars indicate mean values. P values were calculated by two-tailed unpaired Student’s t test (*, P < 0.05; **, P<0.005). (TIF) [file ppat.1009758.s011.tif]
